# The Separate Spheres Model of Gendered Inequality

**DOI:** 10.1371/journal.pone.0147315

**Published:** 2016-01-22

**Authors:** Andrea L. Miller, Eugene Borgida

**Affiliations:** Department of Psychology, University of Minnesota, Minneapolis, Minnesota, United States of America; University of Geneva, SWITZERLAND

## Abstract

Research on role congruity theory and descriptive and prescriptive stereotypes has established that when men and women violate gender stereotypes by crossing spheres, with women pursuing career success and men contributing to domestic labor, they face backlash and economic penalties. Less is known, however, about the types of individuals who are most likely to engage in these forms of discrimination and the types of situations in which this is most likely to occur. We propose that psychological research will benefit from supplementing existing research approaches with an individual differences model of support for separate spheres for men and women. This model allows psychologists to examine individual differences in support for separate spheres as they interact with situational and contextual forces. The separate spheres ideology (SSI) has existed as a cultural idea for many years but has not been operationalized or modeled in social psychology. The Separate Spheres Model presents the SSI as a new psychological construct characterized by individual differences and a motivated system-justifying function, operationalizes the ideology with a new scale measure, and models the ideology as a predictor of some important gendered outcomes in society. As a first step toward developing the Separate Spheres Model, we develop a new measure of individuals’ endorsement of the SSI and demonstrate its reliability, convergent validity, and incremental predictive validity. We provide support for the novel hypotheses that the SSI predicts attitudes regarding workplace flexibility accommodations, income distribution within families between male and female partners, distribution of labor between work and family spheres, and discriminatory workplace behaviors. Finally, we provide experimental support for the hypothesis that the SSI is a motivated, system-justifying ideology.

## Introduction

For more than a decade, the dominant social-psychological approach to studying gendered workplace inequality and discrimination has been to investigate the role of descriptive and prescriptive gender stereotypes (e.g., [1]). Social psychologists have established that when women appear to be too feminine, maternal, communal, or warm, they are regarded as not being competent enough or committed enough to the workplace [2]. This can lead to backlash in the form of fewer recommendations for promotions, lower hiring rates, and less willingness to educate mothers compared to other employees [3–6]. Conversely, when women appear too masculine, agentic, or competent, they are penalized for violating gender norms of femininity [1, 4, 6–10]. Men similarly face backlash in the workplace when they violate gender norms of masculinity and workplace devotion by attempting to engage in caregiving and other forms of domestic labor [10–14].

A common theme in the study of gender is the idea that men and women belong in distinct spheres of society, with men being particularly fit for the workplace and women being particularly fit for the domestic domain. As described above, research on role congruity theory and descriptive and prescriptive stereotypes in the workplace has established that when men and women violate gender stereotypes by crossing spheres, with women pursuing career success and men contributing to domestic labor, they face backlash and economic penalties. Less is known, however, about the types of individuals who are most likely to engage in these forms of backlash and discrimination and the types of situations in which this is most likely to occur. Although the idea of separate-but-equal spheres for men and women has existed for a long time in our culture [15–16], social psychologists have not systematically examined this belief system as a psychological process. We propose that psychological research on gendered behavior and outcomes in society will benefit from supplementing existing research approaches with a model that places the locus of causation on the attitudes and behaviors of the actor doing the discriminating. This approach allows psychologists to examine individual differences in support for the SSI as they interact with situational and contextual forces. In this set of studies, we characterize the SSI as a psychological construct marked by individual differences and motivated system justification tendencies, operationalize the SSI with a new scale measure, and model the SSI as a predictor of several important forms of gender inequality in society. This paper thus presents a new theoretical approach that augments existing approaches to the study of gender in social psychology, as well as a new measurement tool.

Accordingly, we present the Separate Spheres Model, which provides a psychological construct for the belief system known as the separate spheres ideology and predicts specific relationships between individuals’ endorsement of this ideology and other cognitive, attitudinal, and behavioral processes. In particular, the Separate Spheres Model proposes that individual endorsement of the separate spheres ideology interacts with a variety of contextual and situational forces to produce gendered outcomes in society. As a first step toward developing the Separate Spheres Model and examining the contours of the separate spheres ideology, we develop a measure of individuals’ endorsement of this belief system and demonstrate the reliability, convergent validity, and incremental predictive validity of the measure in relation to other contemporary measures of gendered attitudes. We also provide support for the novel hypotheses that the separate spheres ideology predicts attitudes regarding workplace flexibility accommodations, reported income distribution within families between male and female partners, reported distribution of labor between work and family, and reported workplace conduct. Finally, we provide experimental support for the novel hypothesis that the separate spheres ideology is a motivated belief system, taking the form of a system-justifying ideology.

### The Separate Spheres Ideology as a New Theoretical Psychological Construct

The general notion of separate spheres for men and women is deeply ingrained in our culture. Journalists, legal scholars, and social scientists have observed a wide variety of gendered phenomena that seem to be manifestations of the public’s insistence that men and women occupy separate spheres. In contrast, we propose that the separate spheres ideology is a measurable *psychological* construct with individual and situational variability. We posit that by investigating individuals’ endorsement of the separate spheres ideology as an individual difference and modeling the separate spheres ideology as a source of behavioral outcomes, social psychologists and other social scientists may uncover previously unexamined sources of individual and situational variation in the tendency to engage in gendered discrimination and backlash against others and to support policies that exacerbate gendered inequality in society.

Because psychologists have not systematically studied the separate spheres ideology, we borrow from literatures in other fields to develop a working definition of the belief system. We define the separate spheres ideology (SSI) as a belief system that claims that: 1) gender differences in society are innate, rather than culturally or situationally created; 2) these innate differences lead men and women to freely participate in different spheres of society; and 3) gendered differences in participation in public and private spheres are natural, inevitable, and desirable. We conceive of the separate spheres ideology as a single construct composed of these interrelated tenets. Furthermore, in contrast to gender ideology, which is typically defined in terms of one’s *personal identity* with regard to marital and family roles [17–20], the SSI as we define it is a set of beliefs about the proper roles of men and women in society. We outline in more detail several important facets of this belief system as we have conceptualized it and illustrate these facets with examples of various social phenomena observed by other scholars.

#### The SSI contends that gender differences are innate

According to this belief system, the gender differences we observe in society are natural and innate. For example, social psychologists have demonstrated a tendency among some individuals to essentialize differences between men and women, viewing gender categories as immutably distinct [21–23]. Social role theory argues that individuals’ beliefs about differences between men and women originate from observing men and women in different social roles, and that these beliefs then foster real differences in behavior between men and women [24]. Regardless of where gender essentializing tendencies originate, these tendencies constitute one component of support for the SSI.

#### The SSI neglects situational constraints on men’s and women’s behavior

This belief system not only emphasizes the innateness of gender differences in parenting roles and career ambition, but it also deemphasizes the extent to which societal institutions reproduce gender differences and inequality. For example, several researchers in sociology have demonstrated that young men and women in the U.S. have remarkably similar goals in terms of establishing gender-egalitarian families in which male and female partners contribute equally at home; however, structural forms of gender inequality prevent people from creating gender egalitarian household arrangements [25–26]. Other researchers have found that when fathers try to contribute to caregiving duties at home, they are sometimes penalized for using flexibility benefits at work to do so [11, 13]. The SSI deemphasizes these institutional forces that encourage the perpetuation of gender differences in favor of the belief that these gender differences are natural and innate.

#### The SSI posits that gender differences in participation are a matter of personal choice

This belief system assumes that the reason women are underrepresented and under-rewarded in the workplace is that women freely choose to prioritize family over work (a choice that is rooted in women’s innate needs for caregiving, as described above). Print media perpetuate the idea that mothers are freely opting out of working, while ignoring the evidence that shows they are being systematically pushed out of the workplace [27–28]. In contrast to the media image of white, middle- to upper-class women choosing to leave their careers in droves, research suggests that women who leave the workplace when they have children are less common than popularly believed [29–30]. They are also often low-income women who cannot afford the cost of childcare on their low wages [29–30]. Finally, women are frequently lauded for opting out of the workplace entirely to care for their children, but are often punished if they stay at work and make use of flexibility policies [31]. Thus, while there are significant situational and institutional factors that influence the “opt-out” phenomenon, the SSI characterizes this act as a matter of personal choice by individual women.

#### The SSI likens separate gendered spheres with equality

This belief system relies on a separate-but-equal logic that regards women’s homemaking role as equally important and fulfilling as men’s working role, even while insisting that these spheres be segregated by gender. In other words, this is not an ideology that explicitly argues that women are inferior to men (although the reality is that care work in our society is not economically rewarded as much as work in the public sphere). Scholars have observed that individuals are decreasingly willing to report that they view women as inferior to men, and instead are more likely to report resentment toward women who break out of traditional roles [32].

#### The SSI then and now

As these examples suggest, the separate spheres ideology is a concept with several interrelated tenets. It seems that the SSI harms both men and women (although not necessarily in directly comparable ways) by restricting women’s abilities to contribute fully to society and restricting men’s abilities to participate fully in their family lives. The SSI also harms individuals who identify as gay, lesbian, bisexual, and transgender, and individuals who do not subscribe to the gender binary, by failing to recognize the legitimate existence of alternative gender identities, roles, and pairings. We note that our discussion is not meant to draw sharp distinctions between so-called old and new forms of sexism, nor is it meant to suggest that the separate spheres ideology is itself new as a cultural idea. We posit that the SSI continues to be relevant in society today while also reproducing old-fashioned patterns of inequality in employment, politics, law, and the home.

Despite the existence of the separate spheres ideology as a cultural idea in society for so many years, the notion of separate spheres as an ideological belief system has not been examined in psychology. We know quite a bit about the types of perceptions and behaviors that may lead an individual to experience gendered discrimination and backlash, particularly in the workplace, but we know little about individual and situational variation in the tendency to engage in gendered discrimination and backlash against others. In this set of studies, we characterize the SSI as a psychological construct marked by individual differences and motivated system justification tendencies, operationalize the SSI with a new scale measure, and model the SSI as a predictor of several important forms of gender inequality in society. This paper thus presents a new theoretical approach that augments existing approaches to the study of gender in social psychology, as well as a new measurement tool. The first step toward developing the Separate Spheres Model is creating a measure of individuals’ endorsement of the SSI. We now examine how the measurement of the SSI differs from previous work in social psychology.

### The SSI Has Not Been Operationalized in Social Psychology

Although there are no measures of the separate spheres ideology in social psychology, there are a few individual-difference measures that capture other aspects of gendered attitudes and beliefs. These measures are useful for different measurement purposes, and the Separate Spheres Ideology scale presented here is not meant to supplant them. We briefly review here a few of the most frequently used gender-related attitude measures used in empirical social psychology research during the past few decades.

First, the Ambivalent Sexism Inventory [33] measures individuals’ beliefs about the proper relationships between men and women. The Benevolent Sexism subscale measures the belief that women should be treated with protection and paternalism by men (e.g., “Every man ought to have a woman whom he adores.”). The Hostile Sexism subscale measures the belief that women should be subservient to men and overall resentment and hostility toward women who fail to be submissive (“Women seek to gain power by getting control over men.”). These scales also capture positive and negative stereotypes about women (“Women, compared to men, tend to have a superior moral sensibility.”). The Ambivalent Sexism Inventory has been important to theoretical advancements in the study of sexism. For example, the idea that some forms of sexism take on positive tones has been pivotal to psychologists’ understanding of why some women endorse sexist beliefs that disadvantage them [34–35]. While women regularly reject hostile sexism, they often endorse benevolent sexism, which serves to place women on a pedestal and obscure the harmful gender disparities that they face.

A primary difference between the Ambivalent Sexism Inventory and the Separate Spheres Ideology scale is that the former does not (and is not meant to) assess the extent to which individuals endorse the separation of men and women into different spheres of society. Furthermore, the Ambivalent Sexism Inventory primarily captures positive and negative attitudes about women, rather than men. In contrast, the Separate Spheres Ideology scale captures beliefs about the proper social roles for both men and women, and we posit that the ideology serves to police both men and women into their traditional roles. The Separate Spheres Ideology scale draws inspiration from research on Ambivalent Sexism, because it recognizes that sexism is often nuanced and positive in tone. For example, by glorifying women’s natural abilities in caregiving roles, individuals can simultaneously believe that men belong at work and women belong at home and believe that men and women living under this separated system are “equal.”

The Modern Sexism scale [32] measures the extent to which individuals deny the existence of continuing discrimination against women (e.g., “Society has reached the point where women and men have equal opportunities for achievement.”). Similarly, the Gender System Justification scale [35] measures individuals’ belief that, broadly speaking, gender relations in today’s society are fair and just (e.g., “Most policies relating to gender and the sexual division of labor serve the greater good.”). Individuals who score high on gender system justification believe that the gender status quo is as it should be. This belief could either stem from the belief that gender inequality has been eradicated (which is similar to the belief underlying modern sexism) or from the belief that existing gender disparities are themselves just (which is more similar to the third tenet of the SSI as we have conceptualized it). As with the Ambivalent Sexism Inventory, these scales are useful for measuring what they were designed to measure and have been important in the psychological study of gender inequality. However, neither scale addresses individuals’ endorsement of the separation of men and women into different spheres of society. The SSI approach draws inspiration from these scales in that it recognizes the role of the separate-but-equal mentality toward gender in justifying and perpetuating inequality. We propose that individuals who believe that women have innate caregiving propensities that men lack are better able to justify restricting women to the domestic sphere and restricting men out of it. They are also likely to regard this separation as fair and just.

The Old-Fashioned Sexism Scale [32] and the Attitudes Toward Women scale [36] measure broader forms of sexism that tap into some concepts related to the separate spheres ideology, as well as other forms of sexism. For example, the Old-Fashioned Sexism scale includes an item regarding men’s and women’s involvement in the domestic sphere (“When both parents are employed and their child gets sick at school, the school should call the mother rather than the father.”), as well as some items regarding women’s general inferiority to men (e.g., “Women are generally not as smart as men.”). Similarly, the Attitudes Toward Women scale includes some items that relate to gendered social spheres (“Women should be concerned with their duties of childbearing and house tending rather than with desires for professional or business careers.”), as well as many items that capture other types of sexism (e.g., “Intoxication among women is worse than intoxication among men.” “Women should be encouraged not to become sexually intimate with anyone before marriage, even their fiancés.”). Thus, although the Old-Fashioned Sexism scale and the Attitudes Toward Women scale contain a few items related to the concept of separate spheres, they contain many items that tap into different constructs altogether—particularly, forms of sexism that are more outdated. In particular, we conducted a survey in preparation for this research (N = 303) and found that only 5.3% of the sample endorsed sexist attitudes using the Old-Fashioned Sexism scale, and only 6.9% of the sample endorsed sexist attitudes using the Attitudes Toward Women scale. Thus it seems that many of the beliefs represented on these scales are outdated enough that participants will not endorse them on a survey (see also [37]). For this reason, these scales are not included in the analyses described in this paper. The SSI scale draws inspiration from these scales insofar as they tap into beliefs about social roles for women, but the SSI scale diverges conceptually from these scales in that it does not also measure other forms of sexism.

In sum, existing individual-difference measures of gender attitudes in social psychology have inspired our research on the separate spheres ideology. Each of these measures addresses concepts that are somewhat related to the SSI or downstream consequences, but none of them captures the construct of interest here—specifically, the notion that men and women naturally fit in different domains of society and should be restricted to these domains. In Studies 1 through 3, we show that individuals’ endorsement of the SSI is significantly correlated with, but distinct from, scores on benevolent and hostile sexism, gender system justification, and modern sexism. We also show that endorsement of the SSI predicts important outcomes above and beyond these measures.

### The SSI Scale is Distinct from Existing Measures of Gender Attitudes in Sociology and Political Science

Unlike in social psychology, there exist measures in sociology and political science that are meant to capture at least some aspects of individuals’ endorsement of separate spheres for men and women. However, because of the way these measures are designed, their utility for social psychologists is limited. Davis and Greenstein [38] conducted a useful review of separate spheres measures used in sociology. They identified 34 single items that have been used to different degrees in various sociological surveys (e.g., “Women are much happier if they stay at home and take care of their children.”). These items are used in pairs or groups of a few items at a time, and often the combinations of items differ from study to study in ad hoc ways. They are typically used in large-scale nationally representative surveys that measure a wide variety of attributes (e.g., the General Social Survey). The 34 items vary somewhat in the extent to which they capture endorsement of separate spheres, but they are all at least loosely related to this concept. Although Davis and Greenstein [38] state that each of these survey items is reliable and valid, the items have not been validated as scales. The situation is similar in political science, where a primary measure of endorsement of separate spheres is one single-item measure used in the American National Election Study (ANES).

Single survey items are useful for the purpose they are meant to serve; when conducting a large survey in a nationally representative sample, there often isn’t adequate space or time to include full scales for each construct of interest. For social psychologists interested in studying psychological processes, however, these measures are not useful. First, because the separate spheres ideology in particular is so multi-faceted, psychologists must use a multi-item scale to ensure concept validity. Second, by aggregating many items together into one validated scale, researchers increase the reliability of their measures [39], which is particularly important when examining subtle, hard-to-detect psychological phenomena [40]. Finally, psychologists often don’t face the same logistical constraints regarding the length of survey measures that sociologists and political scientists face. When conducting experiments of specific, targeted hypotheses (as opposed to a large survey meant to provide data for many different studies), it is easier to ask participants to complete an entire scale.

For all of these reasons, sociology and political science survey items that measure gender ideology are useful for some purposes, but they are not useful for psychologists hoping to examine the psychological antecedents, processes, and consequences of the SSI. In Studies 1 through 3, we show that individuals’ endorsement of the SSI is significantly correlated with, but distinct from, measures of gender ideology used in sociology and political science. We also show that endorsement of the SSI outperforms these measures in predicting important outcomes.

### Other Attributes Relevant to the Separate Spheres Ideology

One participant attribute that should be related to individuals’ endorsement of the SSI is gender. As discussed above, both men and women can hold sexist attitudes, particularly sexist attitudes that are more nuanced and positive in tone, such as benevolent sexism [34]. For this reason, we do not expect gender differences in SSI scores to overwhelm similarities between men and women. However, because women are disadvantaged economically by their restricted access to the work sphere, women may demonstrate somewhat lower average endorsement of the SSI than men.

Political conservatism should also correspond to endorsement of the SSI. Researchers have identified connections between political conservatism and needs for order and structure, support for tradition, and justification of inequality [41]. These tendencies and values are consistent with a desire to structure society neatly along gendered lines and to reinforce traditional notions of appropriate roles for men and women.

In Studies 1 through 3, we show that individuals’ endorsement of the SSI is significantly correlated with, but distinct from, political ideology and gender. We also show that endorsement of the SSI predicts important outcomes above and beyond political ideology and gender.

Hypothesis 1 (Convergent Validity): Scores on the Separate Spheres Ideology scale will be significantly correlated with scores on benevolent and hostile sexism, gender system justification, modern sexism, gender ideology items from sociology and political science, gender, and political conservatism.

### The SSI Predicts Important Gendered Outcomes in Society

In addition to operationalizing the SSI with a new scale measure, the Separate Spheres Model models the SSI as a predictor of important gendered outcomes in society. One potential outcome of endorsing separate spheres is opposition to the flexibility accommodations that are offered by some American workplaces. Many workplaces are structured around a schedule that assumes each worker is fully available to the company and has no outside life conflicts [10]. Workplace policies that provide flexibility accommodations (e.g., parental leave, tele-commuting, flexible start and end times) allow employees to adapt their schedules so that they can attend to both their careers and their personal and family lives as needed. As such, they have the effect of blurring the boundaries between the work and home spheres, and some of these accommodations help reduce economic inequality for women who have both careers and families to care for. Individuals who endorse separate spheres for men and women may oppose these policies because they have the effect of both encouraging men to participate in family caregiving and encouraging women to invest in their careers. On the other hand, it is possible that, particularly in an American context embodied by individualism and meritocracy ideals, that some individuals recognize gendered inequality in the workplace but still do not think that workplaces should bear the burden of enacting flexibility policies.

Hypothesis 2 (Flexibility Policies): a) Scores on the Separate Spheres Ideology scale will predict opposition to workplace flexibility policies. b) This effect will occur above and beyond the effects of benevolent and hostile sexism, gender system justification, modern sexism, gender, and political conservatism.

A second potential consequence of the SSI is the way in which families are structured. In many families, a woman’s job is regarded as the “second job”; it is often the first to go if the family relocates or decides to live on a single income [42]. It’s possible that individuals who endorse separate spheres for men and women are more likely to have a family income structure in which the man’s income is higher than the woman’s (assuming a cisgender and heterosexual partnership).

Hypothesis 3 (Division of Family Income): a) Scores on the SSI scale will predict traditional family income structure. b) This effect will occur above and beyond the effects of benevolent and hostile sexism, gender system justification, modern sexism, gender, and political conservatism.

A third potential outcome of individual endorsement of the SSI is the extent to which individuals participate in the work and domestic spheres. As described above, society often holds women to higher standards when it comes to domestic responsibilities [16], and society holds men to higher standards when it comes to career devotion [11, 13]. It’s possible that individuals who endorse the SSI structure their own time in gendered ways, such that men spend more time at work and women spend more time on caregiving duties.

Hypothesis 4 (Participation in Each Sphere): Men’s SSI scores will predict working more hours per week. Women’s SSI scores will predict spending more time on caregiving duties.

Finally, a fourth potential outcome of individual endorsement of the SSI is supervisors’ conduct toward their employees in the workplace. Psychologists and legal scholars have documented significant incidents of flexibility stigma and discrimination in the workplace on the basis of employees’ caregiving and family responsibilities [10, 43]. Supervisors who endorse the SSI may be more likely to engage in discriminatory conduct against employees with caregiving responsibilities, because these employees are blurring the boundaries between the home and workplace spheres.

Hypothesis 5 (Discriminatory Conduct in the Workplace): Supervisors’ SSI scores will predict the extent of self-reported discriminatory actions taken against employees with family responsibilities.

### The SSI is a Motivated, System-Justifying Ideology

A signature feature of the SSI is the separate-but-equal logic that underlies it. This is not a belief system that argues for the inferiority of women; rather, it argues that men and women are different in ways that both deserve respect—women for their innate caregiving abilities, and men for their drive to succeed. Accordingly, the Separate Spheres Model posits that the SSI serves a motivated, system-justifying role for individuals who endorse it; individuals who endorse the SSI rely on this belief system to defend, justify, and perpetuate gendered inequality in society.

System justification is the process of rationalizing and defending the status quo, or the system under which one lives [44]. System justification theory argues that individuals experience a powerful motivation to believe that the system they live under is fair and just. The system can be a nation, an employer, a legal institution, or any other structural environment within which a person operates. Research has demonstrated that people will go to great lengths to justify or explain away the injustices that they see in society so that they can maintain their belief that the world is just.

According to system justification theory, individuals vary in the extent to which they are chronically motivated to justify the system [44]. The strength of this motivation also varies from situation to situation. When people experience system threat, the motivation to justify the system becomes stronger [45–46]. System threat can come in a variety of forms. For example, system threat in the national context might occur after reading an article about how the U.S. is facing economic and social decline. Anything that signals a threat to the status quo has the potential to trigger system justification tendencies. Furthermore, the subject matter of the system threat need not match the subject matter of the particular system that the individual defends [46]; system threat in one domain of a person’s life can lead to increased system justification in a different domain.

Many different ideologies and beliefs can serve system-justifying functions. Just as an individual can endorse the gender system justification scale, expressing a broad belief that gender relations in society are fair, one can also rely on one’s political orientation [41], complementary stereotypes about men and women [35], or essentialist explanations for gender differences [47] to justify the gendered status quo. Similarly, the Separate Spheres Model proposes that the SSI serves this system-justifying function for those who endorse it. Research on the status incongruity hypothesis suggests that agentic women and modest men are penalized in the workplace because these penalties serve to defend the gender hierarchy [48–49]. In contrast, the Separate Spheres Model posits that both men and women who cross spheres are penalized in the workplace because they violate gendered social roles, and these penalties serve to defend the tradition of separate, gendered spheres.

The potential system-justifying function of the SSI has important implications for gendered inequality in society. Research has demonstrated that system justification leads people to not only acquiesce to injustices in society, but to actively resist change [50–51]. In order for social scientists to develop effective interventions that have the potential to decrease inequality, we must first understand the extent to which individuals’ resistance to potential interventions is rooted in their psychological motivations to defend the status quo. For example, in the context of environmental policy, people who experienced stronger system justification needs were more likely to deny climate change and oppose pro-environmental policies [52]. However, when these pro-environmental policies were framed as patriotic and consistent with protecting the status quo, rather than altering the status quo, the effects of system justification were eliminated. Thus, the extent to which the SSI takes the functional form of a system-justifying ideology is not a mere matter of academic debate. This functional form has important implications for the extent to which progress toward gender equality is possible, as well as for the specific strategies that may be successful in this pursuit. In Study 3, we show that individuals strengthen their endorsement of the SSI in response to system threat, suggesting that the SSI is a motivated, system-justifying ideology that individuals actively use to defend and perpetuate gendered inequality in society. We also show that the effect of a system threat manipulation dissipates after individuals have expressed their separate spheres beliefs, suggesting that the SSI serves the palliative function of resolving the individual’s elevated need to justify the system.

Hypothesis 6 (System Justification): Participants who are exposed to system threat will have higher SSI scores than participants who are not exposed to system threat. System threat will not have an effect on subsequent measures of policy attitudes after participants complete the SSI scale.

In three studies, comprising seven samples and 1,138 participants, we first validate a scale to measure individuals’ endorsement of the separate spheres ideology. In Studies 1 through 3, we show that attitudes and behaviors that characterize gender inequality are systematically predicted by individual variation in endorsement of the SSI (represented by Hypotheses 1 through 5). In Study 3, we also show that individuals’ endorsement of the SSI varies across situations in systematic ways, namely, by operating as a motivated, system-justifying ideology (represented by Hypothesis 6).

## Study 1

In Study 1, we constructed the Separate Spheres Ideology scale and tested its reliability and validity. We also examined the extent to which the SSI predicts opposition to workplace flexibility policies above and beyond the effects of existing measures of gender attitudes.

### Method

#### Ethics statement

All of the studies described in this article were approved by the University of Minnesota institutional review board (IRB# 1201P08184, 1302P27801, 1404P49425, 1404P49761). We obtained written consent from participants for each study described in this paper.

#### Participants

Participants in Study 1 were 242 undergraduate students (66 men and 176 women) at a large Midwestern University who completed the study for partial course credit.

#### Materials and procedure

Before the study began, we constructed a pool of 73 potential items for the SSI scale. We primarily produced this list by reviewing past literature on separate spheres. Among the 73 items were statements that captured each tenet of the SSI (gender differences are innate, men and women freely divide themselves into separate spheres, and gendered spheres are desirable). The full set of 73 items included equal numbers of items that focused on men and women. The full set of items also included 31 that were reverse-scored.

Participants in Study 1 arrived to the study and completed the 73 SSI items. Participants saw the 73 items in randomized order on a computer screen and responded to each on a 7-point scale (1 = Strongly Disagree, 7 = Strongly Agree). After completing the potential SSI scale items, participants took a 60-second break. They then completed measures of benevolent and hostile sexism [33], gender system justification [35], and modern sexism [32] (see Table 1 for descriptive statistics for these measures in each study). Next, participants completed 34 single-item measures of gender ideology used by sociologists [38] and another two items from the General Social Survey that were not reviewed by Davis and Greenstein [38]. Next, participants completed the gender item from the American National Election Study (2008 Time-Series, pre-election). Participants then indicated their support for two workplace flexibility policies that blur the line between the gendered spheres (“Companies should be required to provide paid leave for new fathers” and “Companies should be required to provide equal paid leave for new mothers and new fathers”). Finally, participants identified their gender and provided their political orientation on three items (general political orientation, social political orientation, and economic political orientation).

**Table 1:** Descriptive statistics for attitudinal variables in each study.

Variable (Study)	*Items*	*M*	*SD*	*Minimum*	*Maximum*	*α*
Benevolent Sexism (1)	11	3.38	0.81	1.00	5.60	.79
Benevolent Sexism (2b)	11	3.17	1.06	1.00	6.00	.89
Hostile Sexism (1)	11	3.04	0.94	1.00	5.64	.88
Hostile Sexism (2b)	11	2.89	1.14	1.00	5.64	.93
Gender System Justification (1)	8	4.42	0.96	1.50	7.00	.77
Gender System Justification (2b)	8	4.23	1.14	1.25	6.63	.84
Modern Sexism (1)	8	3.39	1.00	1.13	6.13	.84
Modern Sexism (2b)	8	3.20	1.22	1.00	6.38	.90
Political Conservatism (1)	1	3.81	1.54	1.00	7.00	---
Political Conservatism (2a)	1	3.05	1.12	1.00	5.00	---
Political Conservatism (2b)	1	3.43	1.61	1.00	7.00	---
Political Conservatism (2c)	1	3.17	1.56	1.00	7.00	---
Political Conservatism (3a)	1	3.25	1.57	1.00	7.00	---
Political Conservatism (3b)	1	3.17	1.60	1.00	7.00	---

### Results

#### Creation of the SSI scale

An analysis of the full set of 73 items revealed that they were highly reliable (Cronbach’s *α* = .88). None of the individual items detracted from the reliability of the full set; the reliability of the set if each item was deleted ranged from α = .87 to α = .88. In order to narrow down the set, we first removed any items that had item-total correlations in the wrong direction; this resulted in nine items being removed from the scale. We then removed any items with low item-total correlations (below 0.1); this resulted in ten more items being removed. Finally, we removed any items with low standard deviations (below 1.0), because these items did not capture enough variability; this resulted in five more items being removed from the scale.

After taking these steps, 49 SSI items remained. The reliability of this set was high (α = .92). As described above, while we conceptualize the SSI in terms of multiple tenets, we consider these tenets to be interrelated and somewhat logically dependent; we expected the scale items, therefore, to cohere into one ideological construct, as opposed to three separate sub-scales. Using exploratory factor analysis with iterated principal factors, we forced the 49 items into one factor. We did this in order to gauge each item’s contribution to the central construct underlying the SSI, so we could select items most closely related to that central construct. After forcing the items into one factor, we chose the items with the highest loadings on that factor, with a few exceptions. Specifically, the reverse-scored items tended to load less strongly on the factor than the items scored in the standard direction; they also reduced the reliability of the set. We felt, however, that it was important to include reverse-scored items in the final scale, in order to reduce acquiescence and response-style bias. We therefore selected the five reverse-scored items with the highest loadings and the ten standard-scored items with the highest loadings (see Appendix A for the final SSI scale). The resulting reliability of this 15-item scale was α = .88 (see Table 2). After reverse-scoring the appropriate items, we calculated each participant’s SSI score by finding the mean of the 15 responses; high scores indicate stronger endorsement of separate spheres. The mean score in the sample was 3.20 (slightly below the midpoint of 4) and the standard deviation was 1.02 (see Table 2 for full summary of psychometric properties).

**Table 2:** Psychometric properties of the Separate Spheres Ideology scale across seven samples.

Statistic	Study 1	Study 2a	Study 2b	Study 2c	Study 2d	Study 3a	Study 3b
Reliability (α)	.88	.88	.91	.91	.91	.92	.90
Mean	3.20	3.28	3.26	3.09	3.03	3.21	3.00
Standard Deviation	1.02	1.04	1.16	1.10	1.10	1.21	1.09
Kolmogorov-Smirnov (*D*)	.07*	.08	.05	.07	.06	.06*	.07*
Shapiro-Wilk (*W*)	.98*	.98	.99	.98	.98*	.98	.98*
Eigenvalue of first factor	---	6.25	7.00	6.62	7.13	7.40	6.68

*p < .05.

#### Hypothesis 1: Convergent validity

In order to test the convergent validity of the 15-item SSI scale, we measured the correlations between the SSI and other contemporary scales related to gendered attitudes. We expected the SSI to be related to benevolent sexism, hostile sexism, gender system justification, and modern sexism. Consistent with our predictions, the SSI was significantly correlated with benevolent sexism (*r* = .66, *p* < .001), hostile sexism (*r* = .59, *p* < .001), gender system justification (*r* = .42, *p* < .001), and modern sexism (*r* = .54, *p* < .001).

Next, we measured the correlations between the SSI and the single-item measures of sexism used primarily in other fields. We predicted that the SSI would be related to single-item measures of gender ideology used in sociology research and the American National Election Study. The results overwhelmingly supported this prediction; of the 37 correlations that we measured, 35 were significant (see Table 3; the first 34 items are sociological survey items reviewed by Davis & Greenstein [38]; the next 2 items are from the General Social Survey but are not reviewed by Davis & Greenstein; the final item is from the ANES). Significant correlations ranged from *ρ* = .21 to *ρ* = .73, and the mean of all correlations was *ρ* = .46.

**Table 3:** Correlations between the Separate Spheres Ideology scale and single-item measures of sexism used primarily in sociology and political science.

Survey Item (paraphrased)	Spearman’s *ρ*
1. Against both the man and woman contributing to income	.38**
2. Men’s job is to earn money, and women’s job is to look after the home	.67**
3. Husband should earn higher pay than wife	.63**
4. If jobs are scarce, the wife shouldn’t work	.54**
5. Husband should be the main breadwinner	.68**
6. When jobs are scarce, men should have more right to a job	.62**
7. It causes problems when women earn more than their husbands	.38**
8. It’s better if the man is the achiever outside the home	.70**
9. Men and women should not be doing each other’s work	.59**
10. A woman’s place is in the home, not the office or shop	.54**
11. A wife who does her job at home doesn’t have time for paid work	.55**
12. Working mothers can’t have as warm relationships with their children	.41**
13. Preschool children suffer when their mothers work	.41**
14. Family life suffers when the woman has a full-time job	.51**
15. A husband should worry if his wife is away overnight for work	.31**
16. The employment of wives leads to juvenile delinquency	.42**
17. Women are happier when they stay home and care for children	.53**
18. What most women really want is a home and children	.59**
19. Being a housewife is just as fulfilling as working for pay	.26**
20. Having a job is not the best way for women to be independent	.00
21. A wife’s most important task is caring for her children	.43**
22. Working wives don’t feel more useful than stay-at-home wives	.09
23. Successful marriages don’t depend on each partner having freedom	.21**
24. Women have to have children in order to be fulfilled	.54**
25. Wives should not expect husbands to help around the house	.44**
26. If the wife works full-time, the husband shouldn’t have to help at home	.32**
27. Men should not share the work around the house with women	.48**
28. Employment of both parents is not necessary to make a living	.28**
29. Even if both husband and wife work, they should not share housework	.42**
30. A wife should support her husband’s career instead of having her own	.62**
31. Parents shouldn’t encourage as much independence in daughters as sons	.49**
32. University education is more important for boys than girls	.46**
33. Would rather have a son than a daughter	.32**
34. Men make better political leaders than women	.73**
35. Women are not emotionally suited for politics	.66**
36. Won’t vote for a woman presidential candidate	.22**
37. Women’s place is in the home	.64**

**p < .001.

Next, we measured the correlations between SSI scores and participants’ political orientation. We measured separately participants’ political orientation in general, on social issues, and on economic issues. We predicted that the SSI would be correlated with political conservatism on all three measures, but the correlation would be stronger for social issues than for economic issues. The results supported these predictions. The SSI was significantly correlated with general political conservatism (*ρ* = .45, *p* < .001), social conservatism (*ρ* = .50, *p* < .001), and economic conservatism (*ρ* = .21, *p* < .001). Furthermore, the correlation between the SSI and social conservatism was significantly stronger than the correlation between the SSI and economic conservatism (*z* = 3.57, *p* < .001).

Finally, we predicted that men would score higher on the SSI scale than women. The results of an independent samples *t*-test confirmed this prediction. Men (*M* = 3.80, *SD* = .87) endorsed the SSI significantly more strongly than women (*M* = 2.98, *SD* = .99, *t*(240) = 5.94, *p* < .001, *Cohen’s d* = .88).

#### Hypothesis 2: Flexibility policies

The Separate Spheres Model posits that individuals’ endorsement of the SSI translates into opposition to flexibility policies that allow people to blur the boundaries between the domestic and work spheres. In order to test this substantive hypothesis, as well as the predictive and discriminant validity of the SSI scale, we measured participants’ opposition to two workplace policies requiring businesses to provide paid paternity leave and requiring equal leave time for mothers and fathers. We predicted that SSI scores would predict opposition to these policies above and beyond the effects of other gender-related attitudes. The results of linear regression models confirmed our predictions.

In the first model, SSI scores predicted opposition to paid paternity leave (*β* = .30, *t* = 4.88, *p* < .001). In a second model, SSI scores continued to predict opposition to paid paternity leave, even when benevolent sexism, hostile sexism, gender system justification, modern sexism, gender, and political conservatism were included as predictors (*β* = .24, *t* = 2.48, *p* < .02; see Table 4). Despite the fact that each of these predictors is significantly correlated with the SSI, the full models predicting opposition to paid paternity leave and opposition to equal parental leave did not suffer from significant multicollinearity. The Variance Inflation Factor for each predictor ranged from 1.22 to 2.59. Furthermore, these other measures failed to predict opposition to paid paternity leave. In the combined model, hostile sexism has a significant coefficient in the wrong direction (see Table 4); however, in a model that uses *only* hostile sexism to predict opposition to paid paternity leave, hostile sexism is not a significant predictor (*β =* .10, *t* = 1.49, *p* = *ns*). This may be the result of a suppressor effect [53–55]. If hostile sexism has both an indirect effect on policy attitudes and a direct effect in the opposite direction, these effects may cancel each other out when hostile sexism is the only predictor in the model. When other relevant predictors are added to the model, they may control for the indirect effects of hostile sexism on policy attitudes, leaving only the direct effect in the opposite direction. If this is the case, whatever effect hostile sexism has on policy attitudes in the theoretically predicted direction is an indirect effect through other relevant attitudes in the model and does not survive as a predictor when SSI scores are introduced.

**Table 4:** The separate spheres ideology predicts opposition to paid paternity leave when controlling for other relevant measures (Study 1).

Variable	*β*	*t*	Bivariate correlation (*ρ*)
Separate Spheres Ideology	.24	2.48*	.29**
Benevolent Sexism	.03	0.38	.20*
Hostile Sexism	-.18	-2.23*	.11
Gender System Justification	.11	1.23	.25**
Modern Sexism	.00	0.02	.19*
Participant Gender (man)	.10	1.52	.19*
Political Conservatism	.12	0.09	.26**

**p < .001;

*p < .05.

Finally, we ran a series of linear regression models to determine whether the SSI scale predicted opposition to paid paternity leave when controlling for the measures most conceptually similar to the SSI scale. The SSI scale continued to predict opposition to paid paternity leave when controlling for each of the 34 gender ideology items used in sociological research (see Table 5). Thus, the SSI scale demonstrated incremental predictive validity and discriminant validity; SSI scores predicted policy attitudes that benevolent sexism, hostile sexism, gender system justification, modern sexism, gender, and political conservatism failed to predict.

**Table 5:** The separate spheres ideology predicts opposition to paid paternity leave when controlling for single-item measures of gender ideology (Study 1).

	SSI Scale		Gender Ideology Item	
Gender Ideology Item^a^	*β*	*t*	*β*	*t*
1	.21	3.21*	.26	4.05*
2	.25	3.08*	.07	0.88
3	.33	4.26*	-.05	-0.69
4	.34	4.66*	-.07	-0.94
5	.32	3.91*	-.03	-0.33
6	.30	3.84*	.00	0.06
7	.34	5.07*	-.10	-1.49
8	.29	3.42*	.01	0.15
9	.26	3.50*	.07	0.90
10	.23	3.31*	.13	1.88
11	.25	3.53*	.11	1.50
12	.27	4.02*	.09	1.40
13	.29	4.26*	.02	0.35
14	.27	3.86*	.07	1.05
15	.29	4.50*	.04	0.68
16	.31	4.54*	-.02	-0.27
17	.28	3.80*	.05	0.65
18	.26	3.44*	.07	0.96
19	.33	5.09*	-.09	-1.42
20	.30	4.90*	.08	1.29
21	.32	4.61*	-.03	-0.50
22	.31	5.08*	.12	1.98*
23	.29	4.70*	.05	0.72
24	.31	4.27*	-.01	-0.19
25	.26	3.94*	.13	1.95
26	.26	4.17*	.16	2.48*
27	.18	2.62*	.27	4.05*
28	.26	4.06*	.13	2.09*
29	.18	2.73*	.31	4.88*
30	.31	4.03*	-.01	-0.16
31	.22	3.30*	.18	2.71*
32	.27	3.97*	.09	1.31
33	.30	4.57*	.01	0.13
34	.27	3.07*	.04	0.43

^a^ Each line depicts the results of a separate linear regression model. Item numbers correspond to the items listed in Table 3. Gender ideology items are coded such that higher scores indicate support for more traditional gender roles.

*p < .05.

SSI scores also predicted opposition to employers providing equal leave time for mothers and fathers (*β* = .27, *t* = 4.29, *p* < .001). In a second model, SSI scores continued to predict opposition to equal leave time for mothers and fathers, even when benevolent sexism, hostile sexism, gender system justification, modern sexism, gender, and political conservatism were included as predictors (*β* = .20, *t* = 2.07, *p* < .04; see Table 6). Furthermore, benevolent sexism, hostile sexism, gender system justification, and modern sexism failed to predict opposition to equal leave time. In the combined model, hostile sexism has a significant coefficient in the wrong direction (see Table 6); however, in a model that uses *only* hostile sexism to predict opposition to paid paternity leave, hostile sexism is not a significant predictor (*β =* .04, *t* = 0.55, *p* = *ns*). Finally, we ran a series of linear regression models to determine whether the SSI scale predicted opposition to equal leave time for mothers and fathers when controlling for the measures most conceptually similar to the SSI scale. The SSI scale continued to predict opposition to paid paternity leave when controlling for each of the 34 gender ideology items used in sociological research (see Table 7). Thus, the SSI scale demonstrated incremental predictive validity and discriminant validity; SSI scores predicted policy attitudes that benevolent sexism, hostile sexism, gender system justification, and modern sexism failed to predict. It appears that endorsement of the separate spheres ideology plays an important role in individuals’ opposition to parental leave policies that alleviate women’s caregiving work and allow men to participate more fully in their families.

**Table 6:** The separate spheres ideology predicts opposition to equal leave time for mothers and fathers when controlling for other relevant measures (Study 1).

Variable	*β*	*t*	Bivariate correlation (*ρ*)
Separate Spheres Ideology	.20	2.07*	.27**
Benevolent Sexism	.08	0.98	.21**
Hostile Sexism	-.24	-3.10*	.05
Gender System Justification	.08	0.86	.22**
Modern Sexism	-.05	-0.55	.15*
Participant Gender (man)	.15	2.27*	.21*
Political Conservatism	.21	2.97*	.28**

**p < .001;

*p < .05.

**Table 7:** The separate spheres ideology predicts opposition to equal leave time for mothers and fathers when controlling for single-item measures of gender ideology (Study 1).

	SSI Scale		Gender Ideology Item	
Gender Ideology Item^a^	*β*	*t*	*β*	*t*
1	.19	2.83*	.21	3.26*
2	.23	2.78*	.05	0.66
3	.32	4.01*	-.08	-1.04
4	.29	4.03*	-.05	-0.71
5	.30	3.59*	-.04	-0.53
6	.32	4.05*	-.08	-1.06
7	.32	4.78*	-.13	-2.01*
8	.34	3.98*	-.11	-1.27
9	.28	3.70*	-.03	-0.33
10	.23	3.14*	.08	1.14
11	.28	3.85*	-.02	-0.26
12	.24	3.54*	.06	0.86
13	.25	3.60*	.04	0.64
14	.29	4.09*	-.04	-0.66
15	.27	4.22*	-.03	-0.40
16	.30	4.44*	-.09	-1.30
17	.23	3.15*	.07	0.94
18	.26	3.46*	.01	0.07
19	.26	4.00*	.03	0.45
20	.27	4.35*	.14	2.24*
21	.27	3.95*	-.02	-0.22
22	.27	4.38*	.07	1.11
23	.25	3.91*	.14	2.17*
24	.28	3.86*	-.03	-0.39
25	.24	3.64*	.07	1.08
26	.25	3.84*	.08	1.30
27	.16	2.35*	.23	3.32*
28	.23	3.51*	.13	2.02*
29	.21	3.05*	.16	2.30*
30	.28	3.57*	-.02	-0.20
31	.20	2.92*	.16	2.30*
32	.27	3.95*	-.00	-0.05
33	.25	3.78*	.06	0.86
34	.22	2.40*	.07	0.79

^a^ Each line depicts the results of a separate linear regression model. Item numbers correspond to the items listed in Table 3. Gender ideology items are coded such that higher scores indicate support for more traditional gender roles.

*p < .05.

## Study 2

In Study 2, we further validated the 15-item SSI scale in four independent samples. Three of these samples were recruited using Mechanical Turk, which has been shown to produce relatively diverse samples and high-quality data, particularly in comparison to student samples [56–57]. It was important to rely primarily on diverse adult samples to validate the SSI scale, rather than student convenience samples. Furthermore, because many of our outcome measures are career-related, we recruited two samples that would be specifically relevant to the employment context. We wanted to ensure that the properties of the SSI scale were consistent among people for whom gendered employment-related decisions are relevant and consequential in their professional lives and who are held accountable for their gender-related decision-making in professional contexts. Study 2c consists of participants who were employed at the time of the study, and 2d consists of participants who were employed in supervisory positions at the time of the study (in other words, these participants had the power to make employment decisions regarding other employees, including hiring, firing, promoting, and approving flexibility accommodations). We examined the reliability, convergent validity, and predictive validity of the SSI scale across these samples. We also examined the extent to which the SSI predicts important real-life outcomes related to gender inequality above and beyond the effects of existing measures of gender attitudes.

## Study 2a

### Method

#### Participants

Participants in Study 2a were 64 undergraduates (26 men and 38 women) at a large Midwestern University who completed the study in April 2012 for partial course credit.

#### Materials and procedure

Participants in Study 2a began the study by completing the 15-item SSI scale (see Appendix A). Participants then identified their gender and provided their political orientation on a 7-point scale.

### Results

#### Reliability and psychometric characteristics

In order to further validate the SSI scale in Study 2, we first analyzed the reliability and other psychometric properties of the scale. The scale proved to be reliable (*α* = .88; see Table 2 for the psychometric properties of the scale in each study). The mean score (*M* = 3.28) was slightly below the scale midpoint of 4. This finding is consistent with our expectations, because each of our samples produced a mean political ideology that was slightly to the left of Moderate (see Table 1 for descriptive statistics). Next, we conducted factor analysis using the SSI scale items. Using exploratory factor analysis with iterated principal factors, we left the scale items free to load on any number of factors. We did this in order to verify that the scale items freely loaded onto a single factor. As predicted, a single factor emerged as the sole dominant factor in the model (see Table 2 for eigenvalues; see also S1 File for scree plots from each sample).

#### Hypothesis 1: Convergent validity

Next, we examined the convergent validity of the SSI scale by examining the extent to which scores on the scale correlate with theoretically relevant variables. As in Study 1, we predicted that the SSI would be correlated with political conservatism. The results supported this prediction (*ρ* = .39, *p* < .01).

Finally, we predicted that men would score higher on the SSI scale than women. The results of an independent samples *t*-test confirmed this prediction. Consistent with the results from Study 1, men endorsed the SSI significantly more strongly than women (see Table 8).

**Table 8:** Men endorse the separate spheres ideology more strongly, on average, than women.

Study	Men Mean (SD)	Women Mean (SD)	Mean Difference	df	*t*	*Cohen’s d*
1	3.80 (.87)	2.98 (.99)	0.82	240	5.94**	0.88
2a	3.91 (.92)	2.85 (.90)	1.06	62	4.58**	1.16
2b	3.65 (1.03)	2.93 (1.16)	0.72	148	4.01**	0.66
2c	3.40 (.98)	2.77 (1.12)	0.63	144	3.60**	0.60
2d	3.31 (1.10)	2.76 (1.04)	0.55	151	3.18*	0.53
3a	3.69 (1.11)	2.79 (1.14)	0.90	132	4.58**	0.80
3b	3.26 (1.15)	2.75 (.95)	0.51	245	3.81**	0.48

**p < .001;

*p < .05.

## Study 2b

### Method

#### Participants

Participants in Study 2b were 150 adults (68 men and 82 women) residing across the United States who were recruited using Amazon.com’s Mechanical Turk service in March 2012. The participants ranged from 18 to 79 years of age, with a mean of 34.83 and a standard deviation of 12.56. The sample included participants from 38 states and the District of Columbia. Participants completed the study for $3.00 in compensation.

#### Materials and procedure

Participants in Study 2b volunteered for the study on the Mechanical Turk website. After providing consent, participants first completed the SSI scale. Next, participants completed measures of benevolent and hostile sexism [33], gender system justification [35], and modern sexism [32]. Participants then indicated their support for each of nine different workplace policies designed to address work-life boundaries and gender inequality in the workplace (e.g., flexible start and end times for employees; the ability to work from home). Next, participants indicated their own income and their partners’ (if any) income. Finally, participants identified their gender and their partners’ (if any) gender and provided their political orientation on a 7-point scale.

### Results

#### Reliability and psychometric characteristics

As in Study 2a, the SSI scale was reliable (*α* = .91; see Table 2), and it had a mean score of 3.26 (slightly below the scale midpoint). This finding is consistent with our expectations, because each of our samples produced a mean political ideology that was slightly to the left of Moderate (see Table 1). This finding is also consistent with past work showing that Mechanical Turk samples tend to be slightly liberal [56].Using exploratory factor analysis of the scale items with iterated principal factors, a single factor once again emerged as the sole dominant factor in the model (see Table 2 and S1 File).

#### Hypothesis 1: Convergent validity

Next, we examined the extent to which scores on the scale correlate with contemporary measures of gendered attitudes. We expected the SSI to be related to benevolent sexism, hostile sexism, gender system justification, and modern sexism. Consistent with our predictions, the SSI was significantly correlated with benevolent sexism (*r* = .65, *p* < .001), hostile sexism (*r* = .65, *p* < .001), gender system justification (*r* = .58, *p* < .001), and modern sexism (*r* = .61, *p* < .001). These results replicate the findings from Study 1.

Next, we measured the correlation between the SSI and political orientation. As in the previous studies, the results supported our prediction that SSI scores would be significantly correlated with conservatism (*ρ* = .46, *p* < .001).

Finally, we predicted that men would score higher on the SSI scale than women. The results of an independent samples *t*-test confirmed this prediction. Men endorsed the SSI significantly more strongly than women (see Table 8).

#### Hypothesis 2: Flexibility policies

The Separate Spheres Model posits that individuals’ endorsement of the SSI translates into opposition to flexibility policies that allow people to blur the boundaries between the domestic and work spheres. In Study 2b, we predicted that SSI scores would correlate with opposition to nine specific workplace policies. The results strongly supported these predictions (see Table 9). The correlations ranged from *ρ* = .25 to *ρ* = .41, and all were statistically significant.

**Table 9:** Correlations between the Separate Spheres Ideology scale and opposition to workplace flexibility and equality policies.

Policy (Study)	Spearman’s *ρ*
Paid paternity leave for new fathers (2b)	.30**
Paid paternity leave for new fathers (3a)	.50**
Education programs for supervisors about biases against parents (2b)	.41**
Education programs for supervisors about biases against parents (3a)	.40**
Periodic self-audits of family responsibilities discrimination (2b)	.36**
Periodic self-audits of family responsibilities discrimination (3a)	.41**
Flexible start and end times (2b)	.36**
Flexible start and end times (3a)	.23*
Requirement of equal paid leave time for mothers and fathers (2b)	.31**
Requirement of equal paid leave time for mothers and fathers (3a)	.55**
Zero tolerance of family responsibilities discrimination (2b)	.36**
Zero tolerance of family responsibilities discrimination (3a)	.39**
Work-from-home options for parents (2b)	.33**
Work-from-home options for parents (3a)	.15
System for parents to swap shifts when needed (2b)	.25*
System for parents to swap shifts when needed (3a)	.21*
Extra training for employees returning from extended leave (2b)	.32**
Extra training for employees returning from extended leave (3a)	.34**
Workplace flexibility in general (2c)	.20*

**p < .001;

*p < .05.

Next, we examined the effect of SSI scores on opposition to each flexibility policy while controlling for benevolent sexism, hostile sexism, gender system justification, modern sexism, political conservatism, and gender. A series of linear regression models revealed that none of the variables were consistently significant predictors of the nine flexibility policies. Of the five gender-related scales, the SSI was a significant predictor of opposition to a zero-tolerance discrimination policy (*β* = .26, *p* < .05), and none of the other scales were significant predictors of this policy. Modern sexism was a significant predictor of opposition to a system for parents to swap shifts (*β* = .30, *p* < .05) and opposition to extra training for employees returning from leave (*β* = .40, *p* < .01), and none of the other scales were significant predictors of these policies. Future research is needed to examine why these results were not as consistent as they were in Study 1, but the most likely explanation is that the sample size in Study 2b was smaller than that of Study 1 and therefore had less power.

#### Hypothesis 3: Distribution of family income

The Separate Spheres Model posits that individuals’ endorsement of the SSI relates to how they structure their families and careers. We predicted that individuals’ SSI scores would correspond to the way in which income was distributed within their families. In order to test this hypothesis, we measured participants’ income relative to their partners’ in Study 2b. We examined SSI scores in relation to the likelihood of living in a family with a traditional income structure (an arrangement in which a male partner has a higher income than a female partner); thus, only participants with partners of a different gender were included in this analysis. Men who indicated that they made more money per year than their partners and women who indicated that they made less than their partners were coded as traditional families (1). Men who indicated that they made less than their partners and women who indicated that they made more than their partners were coded as non-traditional families (0). We predicted that SSI scores would predict traditional family income, above and beyond the effects of other relevant attitudes and attributes.

The results of two logit regression models confirmed our predictions. In the first model, SSI scores predicted traditional family income (*B* = .53, *SE* = .17, *Wald* = 10.21, *p* < .001). In a second model, SSI scores continued to predict traditional family income, even when benevolent sexism, hostile sexism, gender system justification, modern sexism, political conservatism, age, education, and gender were included as predictors (*B* = .75, *SE* = .33, *Wald* = 5.13, *p* < .03; see Table 10). In other words, endorsement of the separate spheres ideology predicts the likelihood that an individual’s family will have a traditional income structure, and this relationship exists even when controlling for existing measures of sexism, ideology, and related demographic characteristics. This is yet further evidence that the SSI matters for important life outcomes, not only for predicting attitudes on other psychological scale measures. Furthermore, this result once again demonstrates the incremental predictive validity of the SSI scale.

**Table 10:** Among heterosexual couples, the separate spheres ideology predicts the likelihood that the male partner makes more money than the female partner (Study 2b).

Variable	*B*	*SE*	*Wald*	Bivariate correlation (*ρ*)
Separate Spheres Ideology	0.75	0.33	5.13*	.27*
Benevolent Sexism	-0.37	0.28	1.68	.14
Hostile Sexism	-0.17	0.30	0.32	.24*
Gender System Justification	0.16	0.27	0.35	.25*
Modern Sexism	0.04	0.32	0.02	.24*
Political Conservatism	-0.12	0.15	0.59	.16
Age	0.04	0.02	4.25*	.16
Education	-0.19	0.17	1.24	-.10
Participant Gender (man)	1.98	1.44	2.24	.44**

**p < .001;

*p < .05.

It is important to note that the causal direction of the relationship between endorsement of the SSI and family income is not yet clear. It may be that endorsement of the SSI leads individuals to structure their families in traditional ways, it may be that individuals justify their traditional family arrangements by endorsing the SSI, or it may be that these two phenomena feed back on each other in a reciprocal way. It may also be the case that different causal relationships exist for different individuals.

## Study 2c

### Method

#### Participants

Participants in Study 2c were 146 adults (74 men and 72 women) residing across the United States who were recruited on Mechanical Turk in May 2013. All participants in Study 2c were employed at the time of the study. The participants ranged from 19 to 68 years of age, with a mean of 32.62 and a standard deviation of 10.94. Participants completed the study for $4.00 in compensation.

#### Materials and procedure

Participants in Study 2c volunteered for the study on Mechanical Turk. They were allowed to participate in the study if they indicated that they were currently employed in a job outside of Mechanical Turk. After confirming their employment status, participants first completed the SSI scale. Next, they indicated their overall attitudes toward workplaces providing flexibility accommodations to their employees. Finally, participants identified their gender and provided their political orientation on a 7-point scale.

### Results

#### Reliability and psychometric characteristics

As in the previous studies, the SSI scale was reliable (*α* = .91; see Table 2), and it had a mean score of 3.08. Using exploratory factor analysis of the scale items with iterated principal factors, a single factor once again emerged as the sole dominant factor in the model (see Table 2 and S1 File).

#### Hypothesis 1: Convergent validity

Next, we examined the convergent validity of the SSI scale by examining the extent to which scores on the scale correlate with theoretically relevant variables. As in the previous studies, we predicted that the SSI would be correlated with political conservatism. The results supported this prediction (*ρ* = .44, *p* < .001).

Finally, we predicted that men would score higher on the SSI scale than women. The results of an independent samples *t*-test confirmed this prediction. Men endorsed the SSI significantly more strongly than women (see Table 8).

#### Hypothesis 2: Flexibility policies

The Separate Spheres Model posits that individuals’ endorsement of the SSI translates into opposition to workplace flexibility policies. In Study 2c, we predicted that SSI scores would correlate with negative attitudes toward workplace flexibility accommodations in general. The results supported this prediction (*ρ* = .20, *p* < .02; see Table 9). Thus, in a sample of adults for whom employment-related policies are particularly relevant, the relationship between the SSI and policy attitudes remained robust.

## Study 2d

### Method

#### Participants

Participants in Study 2d were 153 adults (75 men and 78 women) residing across the United States who were recruited on Mechanical Turk in September and October 2014. All participants in Study 2d were employed at the time of the study and held supervisory positions at work. We screened participants using a self-report question (“We consider you a supervisor for this study if a major part of your job is managing and overseeing the work of other employees (for example, if you hire, fire, or promote employees, or if you have the power to approve employees' time off).”). We then followed up with open-ended questions in which participants provided detailed information about the nature of their supervisory work. This procedure allowed us to be more certain that each participant truly was a supervisor at work, while also maintaining the anonymity of the study. The participants ranged from 19 to 60 years of age, with a mean of 33.75 and a standard deviation of 8.39. Participants completed the study for $2.00 in compensation.

#### Materials and procedure

Participants in Study 2d volunteered for the study on Mechanical Turk. Participants were allowed to participate in the study if they were currently employed in a job and held a supervisory position. The study took place in two sessions. In the first session, participants completed the SSI scale and provided their gender and a variety of other demographic information. In the second session, which took place at least five days later for each participant, participants were presented with fourteen workplace behaviors that supervisors might engage in toward their employees (e.g., “I fired an employee after it became clear that his or her family was interfering with his or her work too much”; “I called an employee rude names because I felt that his or her family responsibilities were interfering with his or her job”). Each of the behaviors measured in the study was identified by observing examples from a combination of sources: stories about flexibility stigma in the news, court cases based on family responsibilities discrimination, and data from a pilot study that used an employee sample. The pilot study asked employees to describe the work-life conflicts they had experienced at work, and in their open-ended responses, some participants described experiencing discrimination at work. For each type of behavior, participants indicated whether they had ever engaged in the behavior on a binary measure, as well as how frequently they had engaged in the behavior. Behavior frequency was measured on a 9-point scale (1 = Never, 2 = Less than once per year, 3 = About once per year, 4 = A few times per year, 5 = About once per month, 6 = A few times per month, 7 = About once per week, 8 = A few times per week, 9 = Almost daily).

### Results

#### Reliability and psychometric characteristics

As in the previous studies, the SSI scale was reliable (*α* = .91; see Table 2), and it had a mean score of 3.03. All other scale properties were consistent with the previous studies (see Table 2 and S1 File).

#### Hypothesis 1: Convergent validity

**Fig 1:**
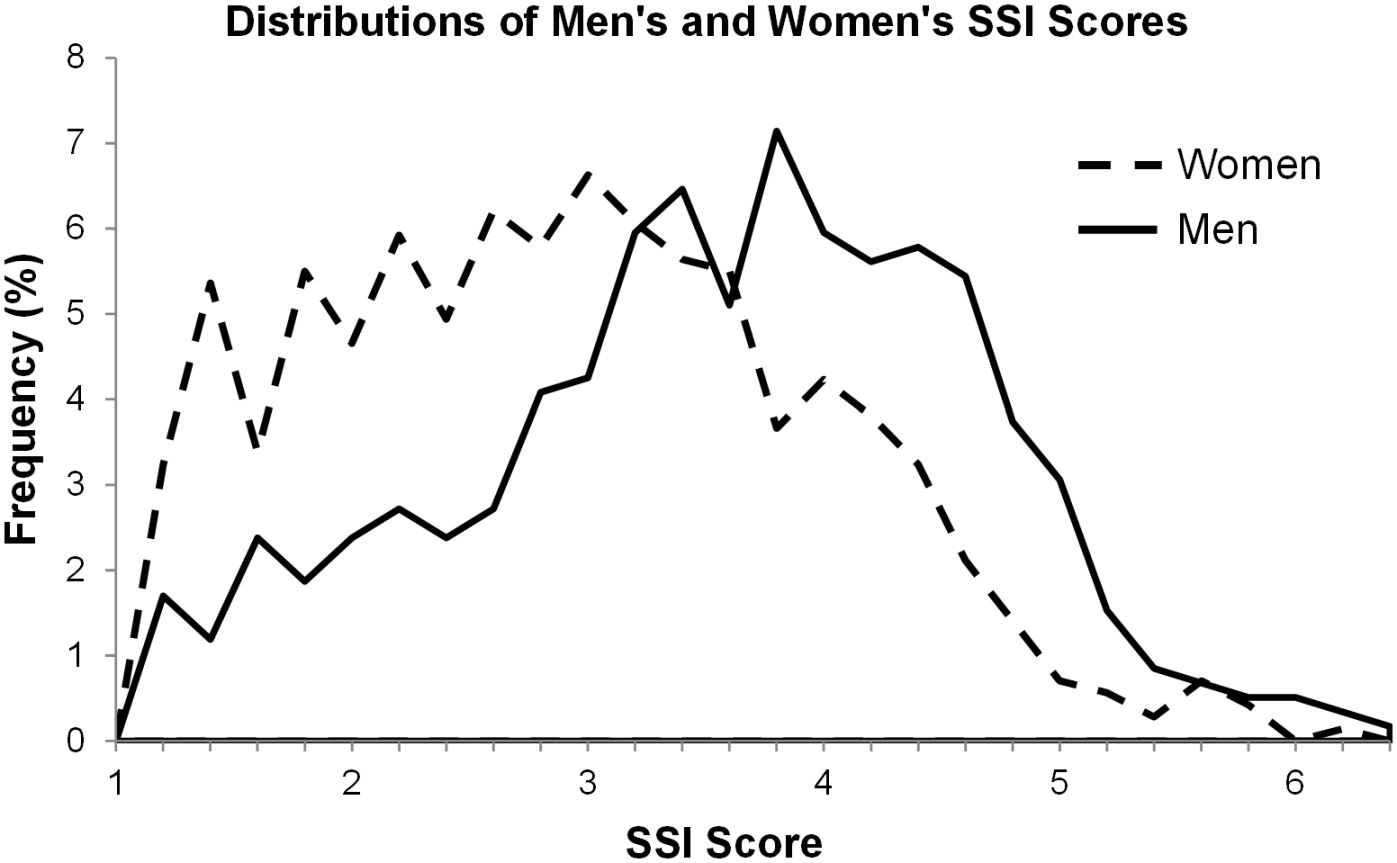
Men’s and Women’s SSI Scores. This histogram includes pooled data from all seven samples. Bins are 0.2 wide.

Next, we examined the convergent validity of the SSI scale by examining the extent to which scores on the scale correspond to theoretically relevant variables. We predicted that men would score higher on the SSI scale than women. The results of an independent samples *t*-test confirmed this prediction. Consistent with the results from the previous studies, men endorsed the SSI significantly more strongly than women (see Table 8). Note that across the four samples in Study 2, the mean gender difference in SSI scores was between about one-half and one full point on the 7-point scale. The standard deviations among men’s and women’s SSI scores were similar to each other in each sample and quite consistent across samples (approximately equal to 1.0). Thus, while there was a consistent gender difference in SSI scores in the predicted direction, it’s also important to note that the differences within men and within women (represented by the standard deviations) were at least as large as the differences between men and women (see Fig 1).

#### Hypothesis 5: Discriminatory conduct in the workplace

The Separate Spheres Model posits that supervisors’ endorsement of the SSI relates to their workplace conduct. We predicted that individuals’ SSI scores would correspond to the extent to which they reported engaging in discriminatory conduct against their employees who had family caregiving responsibilities. We use the word “discriminatory” in the narrow legal sense here, meaning that the participants took these actions against their employees “because of” the employees’ family responsibilities. In order to test this hypothesis, we presented participants with fourteen types of discriminatory or stigmatizing workplace conduct that supervisors might engage in. Participants reported the frequency of engaging in each behavior. Using linear regression, we found that for nine of the fourteen behaviors, supervisors’ SSI scores predicted the frequency of engaging in discriminatory acts against employees with caregiving responsibilities (see Table 11). These findings are striking because they indicate once again that the SSI scale does not merely predict other relevant attitude measures, but important real-world conduct. To the extent that there is a relationship between the SSI scale and employment discrimination against workers with caregiving responsibilities, it will be an important research tool for examining family responsibilities discrimination. Furthermore, this study presents a conservative test of the hypothesis, as the ability to detect the hypothesized relationships depends on supervisors’ willingness to report their own workplace discrimination. The actual frequency of the behaviors measured here is likely higher than the data indicate, suggesting that we were able to identify significant relationships despite a restriction of range in the dependent variable.

**Table 11:** The separate spheres ideology predicts supervisors’ self-reported frequency of discrimination against employees with family caregiving responsibilities (Study 2d).

Discriminatory Conduct^a^	*β*	*t*
Reconsidered a promotion that employee was going to receive	.13	1.65
Talked to employee about inadequate commitment	.21	2.62*
Talked to employee about inadequate performance	.18	2.25*
Rearranged employees’ work assignments	.12	1.51
Terminated employee	.20	2.52*
Terminated employee or asked employee to quit while on leave	.19	2.42*
Convinced employee not to take time off or change schedule	.12	1.44
Prevented employee from taking time off or changing schedule	.19	2.38*
Acted angry with employee	.19	2.33*
Checked on employee to verify reasons for absence	-.03	-0.31
Called employee rude names	.21	2.65*
Demoted employee	.10	1.26
Reduced employee’s hours	.21	2.64*
Acted cold and distant with employee	.25	3.11*

^a^ Each line depicts the results of a separate linear regression model.

*p < .05.

It is once again important to note that the causal direction of the relationship between endorsement of the SSI and discriminatory workplace conduct is not clear. It may be that endorsement of the SSI leads individuals to engage in discriminatory conduct, it may be that individuals justify their discriminatory conduct by endorsing the SSI, or it may be that these two phenomena feed back on each other in a reciprocal way. It may also be the case that different causal relationships exist for different individuals.

## Study 3

In Study 3, we further validated the SSI scale in two independent samples and demonstrated that its psychometric properties remained consistent. We also experimentally examined systematic situational variation in endorsement of the separate spheres ideology and demonstrated that the SSI functions as a system-justifying ideology.

## Study 3a

### Method

#### Participants

Participants in Study 3a were 134 adults (62 men and 72 women) recruited on Mechanical Turk in August 2012. All participants were over 18 years of age and resided in the United States. Participants completed the study for $2.50 in compensation.

#### Materials and procedure

Participants in Study 3a volunteered for the study on Mechanical Turk. In order to manipulate system threat, participants first read what they thought was an excerpt from a newspaper article discussing the state of the nation. Participants were randomly assigned to one of two conditions. In the *system-threat* condition, the newspaper excerpt discussed the decline of social, economic, and political conditions in the U.S. In the *no-threat* condition, the newspaper excerpt discussed the stability and success of social, economic, and political conditions in the U.S. (both excerpts were taken from Kay, Jost, & Young [45]; see S2 File for the text of the stimuli). After exposure to the newspaper manipulation, participants completed the Separate Spheres Ideology scale. Next, participants indicated their support for each of nine different workplace policies designed to address work-life boundaries and gender inequality in the workplace (the same policies used in Study 2b). Next, participants indicated how many hours per week they worked and how much caregiving responsibility they had on a 7-point scale (1 = I don’t have any dependents to take care of, 2 = I take on very few of the caregiving responsibilities for my dependents, 3 = I have some caregiving responsibilities for my dependents, 4 = I share caregiving responsibilities about equally with a partner, 5 = I take on the majority of caregiving responsibilities for my dependents, 6 = I take on almost all of the caregiving responsibilities for my dependents, 7 = I am solely responsible for my dependents). For the purposes of this question, dependents included children, aging family members, adults with disabilities, and any others needing care. Finally, participants identified their gender and provided their political orientation on a 7-point scale.

### Results

#### Reliability and psychometric characteristics

In order to further validate the SSI scale in Study 3, we first analyzed the reliability and other psychometric properties of the scale. The scale proved to be quite reliable (*α* = .92; see Table 2), and it had a mean score of 3.21. Using exploratory factor analysis of the scale items with iterated principal factors, a single factor once again emerged as the sole dominant factor (see Table 2 and S1 File).

#### Hypothesis 1: Convergent validity

Next, we examined the convergent validity of the SSI scale by examining the extent to which scores on the scale correlate with theoretically relevant variables. As in Studies 1 and 2, we predicted that the SSI would be correlated with political conservatism. The results supported this prediction (*ρ* = .53, *p* < .001).

We also predicted that men would score higher on the SSI scale than women. The results of an independent samples *t*-test confirmed this prediction. Consistent with the results from the previous studies, men endorsed the SSI significantly more strongly than women (see Table 8).

#### Hypothesis 2: Flexibility policies

The Separate Spheres Model predicts that individuals’ endorsement of the SSI translates into opposition to workplace policies that blur the line between the gendered spheres. In Study 3a, we predicted that SSI scores would correlate with opposition to nine specific workplace policies. The results strongly supported these predictions (see Table 9). The correlations ranged from *ρ* = .15 to *ρ* = .55, and all but one were statistically significant. This finding replicates the results of Studies 1 and 2 and demonstrates that the relationship between the SSI and important policy attitudes is robust.

#### Hypothesis 4: Participation in gendered spheres

Next, the Separate Spheres Model predicts that individuals’ endorsement of the SSI corresponds to their everyday participation in the gendered spheres. We measured work and caregiving responsibilities in Study 3a to assess how much time each individual participated in the work and domestic spheres, respectively. We hypothesized that participants’ SSI scores would predict these important real-life outcomes. Specifically, we predicted that men with high SSI scores would work more hours per week, but this would not be true for women. We also predicted that women with high SSI scores would spend more time caregiving, but this would not be true for men. The results of four linear regression models confirmed these predictions. Men’s SSI scores predicted the number of hours they worked per week (*β* = .27, *p* < .04), but women’s SSI scores did not predict the number of hours they worked per week (*β* = .09, *p* = ns). Conversely, women’s SSI scores predicted the amount of caregiving responsibilities they had (*β* = .31, *p* < .01), but men’s SSI scores did not predict the amount of caregiving responsibilities they had (*β* = .02, *p* = ns).

Next, we pooled men and women together into each regression model to determine whether there was a significant interaction between gender and SSI scores in predicting each outcome. The results of the first linear regression revealed that there was not a significant interaction between gender and SSI scores in predicting the number of hours worked per week (*β* = .28, *p* = ns). This indicates that although men’s SSI scores significantly predicted their work hours and women’s SSI scores did not significantly predict their work hours (as we hypothesized), the difference between these two effects was not significant. A second linear regression model revealed that there was a nearly significant interaction between gender and SSI scores in predicting caregiving responsibilities (*β* = -.58, *p* = .05). Although this interaction was on the cusp of significance, it suggests that the effect of women’s SSI scores in predicting their caregiving responsibilities was higher than the effect of men’s SSI scores in predicting their caregiving responsibilities. Taken together, all of these findings demonstrate the potentially important role of individuals’ endorsement of the SSI in gendered life outcomes. Furthermore, they show that individuals’ SSI scores predict important life outcomes beyond simply their attitudes on other psychological scale measures.

Next, we broke up the caregiving variable in order to more closely examine the role of SSI scores in predicting the time women spend on caregiving. First, we created a binary variable in which women who did not have dependents were coded as 0 and women who had dependents were coded as 1. We conducted a logit regression in order to determine whether SSI scores predicted the likelihood of having dependents to care for. The results revealed that women’s SSI scores significantly predicted their likelihood of having dependents (*B* = .44, *SE* = .23, *Wald* = 3.86, *p* < .05). Next, we took only the group of women who had dependents and conducted a linear regression in order to determine whether SSI scores predicted the *amount* of caregiving these women did. The results revealed a marginally significant relationship between SSI scores and caregiving responsibilities among women who have dependents (*β* = .29, *p* < .07). It seems that women’s endorsement of the SSI corresponds to whether they have dependents to care for, and it may also be related to the amount of caregiving women do once they have dependents. In contrast, men’s SSI scores are unrelated to the amount of time they spend on caregiving.

Taken together, these findings suggest that one’s endorsement of the separate spheres ideology predicts greater day-to-day participation in the sphere traditionally associated with one’s gender. It is once again important to note that the causal direction of the relationship between endorsement of the SSI and everyday participation in work and caregiving is not yet clear. It may be that endorsement of the SSI leads individuals to structure their time in gendered ways, it may be that individuals justify their gendered sphere participation by endorsing the SSI, or it may be that these two phenomena feed back on each other in a reciprocal way. Future research to tease apart these relationships should be conducted.

#### Hypothesis 6: System justification

**Fig 2:**
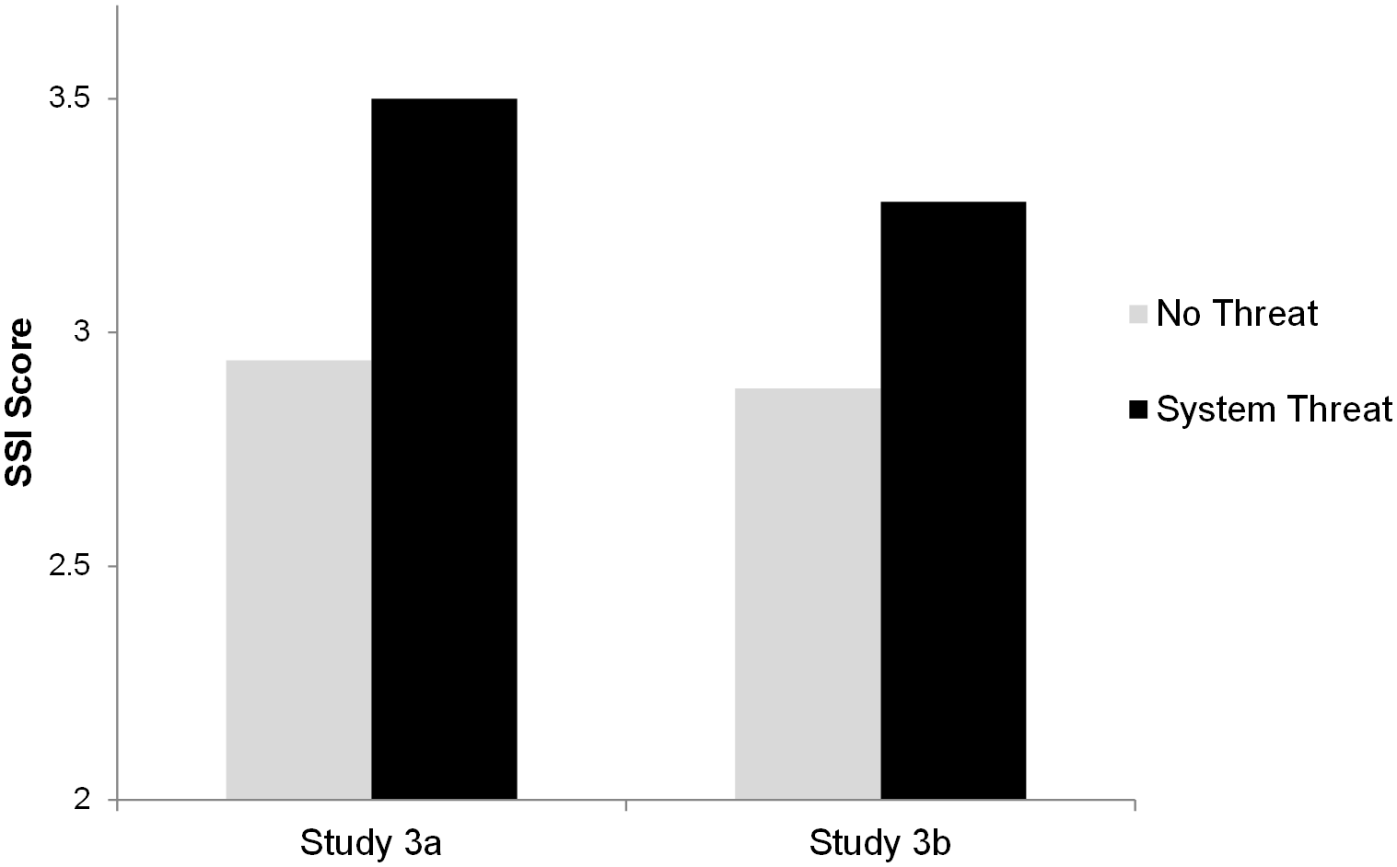
Scores on the Separate Spheres Ideology scale increase in response to system threat (Study 3).

Finally, the Separate Spheres Model posits that the SSI is a system-justifying ideology. We used the SSI scale to test the novel prediction that participants who were exposed to system threat would endorse the separate spheres ideology more strongly than those who were not exposed to system threat. The results of an independent samples *t*-test confirmed this prediction (see Fig 2). In Study 3a, participants in the *system-threat* condition had significantly higher SSI scores (*M* = 3.50, *SD* = 1.29) than participants in the *no-threat* condition (*M* = 2.94, *SD* = 1.07, *t*(132) = 2.71, *p* < .01, *Cohen’s d* = .47). A linear regression further revealed that the system threat manipulation increased SSI scores even when controlling for the effects of political conservatism and gender (*β* = .20, *p* < .01). In other words, as predicted, participants responded to system threat by strengthening their support for the idea that men and women should occupy separate spheres in society.

Next, we examined the proposition that endorsement of the SSI serves a palliative function for individuals with system-justifying needs. We predicted that after participants had completed the SSI scale, thereby expressing their beliefs about gender roles, the system threat manipulation would no longer have an effect on subsequent attitude measures. In a series of linear regression models, we examined the effect of the system threat manipulation on each of the nine measures of opposition to workplace flexibility policies (which were measured immediately after the separate spheres ideology was measured) while controlling for political conservatism and gender. The results supported our predictions, revealing that the system threat manipulation had no significant effect on any of the policy attitudes (see Table 12). Taken together, these findings support our contention that the separate spheres ideology serves a system-justifying function. They suggest that in response to system threat, participants increased their support for the separate spheres ideology in an attempt to justify the gender-role status quo. The results also suggest that the SSI successfully served a palliative function, helping participants to resolve their heightened need for system justification, as the effect of system threat dissipated once participants had been given the chance to express their separate spheres ideology.

**Table 12:** The effects of system threat on opposition to workplace flexibility policies after participants completed the Separate Spheres Ideology scale (Study 3a).

Policy^a^	*β*	*t*
Paid paternity leave for new fathers	.11	1.42
Education programs for supervisors about biases against parents	.07	0.84
Periodic self-audits of family responsibilities discrimination	.03	0.39
Flexible start and end times	.13	1.54
Requirement of equal paid leave time for mothers and fathers	.09	1.14
Zero tolerance of family responsibilities discrimination	.16	1.93
Work-from-home options for parents	.01	0.17
System for parents to swap shifts when needed	.07	0.85
Extra training for employees returning from extended leave	.08	0.95

^a^ Each line depicts the results of a separate linear regression model, controlling for political conservatism and gender.

## Study 3b

### Method

#### Participants

Participants in Study 3b were 249 adults (126 men, 121 women, and 2 who declined to identify their gender) residing across the United States who were recruited on Mechanical Turk in January and February 2013. The participants ranged from 18 to 71 years of age, with a mean of 31.69 and a standard deviation of 11.49. Participants completed the study for $3.00 in compensation.

#### Materials and procedure

Participants in Study 3b volunteered for the study on Mechanical Turk. Participants were asked to read the admissions essay of a college applicant and then provide a series of ratings and recommendations regarding the applicant. Participants were randomly assigned to one of four essay conditions. In each case, the author of the essay was a high school student with a 3.3 grade-point average. In the *system-threat* condition, the student stated that his family could not afford to pay for a college education and lamented the fact that the American Dream seemed not to be true. In the *no-threat* condition, the student stated that through hard work, his family had saved enough money to afford a college education and the American Dream seemed to be alive and well (see S2 File for the text of the stimuli). The two remaining filler conditions were included in the study in order to prevent Mechanical Turk workers from posting information in online forums and discovering that the cover story for the study was untrue (a problem that we have experienced in the past). Participants in these filler conditions received different study stimuli and their data were not meant to be analyzed along with the test conditions. Thus, a total of 123 adults (61 men, 61 women, and 1 participant who declined to provide a gender) participated in the test conditions. After the essay manipulation, participants provided recommendations regarding the student’s admission to a four-year college and ratings of the student’s abilities and potential, in order to enhance the cover story for the study. Finally, participants completed the Separate Spheres Ideology scale, identified their gender, and provided their political orientation on a 7-point scale.

### Results

#### Reliability and psychometric characteristics

We first analyzed the reliability and other psychometric properties of the scale. Once again, the scale proved to be quite reliable (*α* = .90; see Table 2), and it had a mean score of 3.00. Using exploratory factor analysis of the scale items with iterated principal factors, a single factor once again emerged as the sole dominant factor (see Table 2 and S1 File).

#### Hypothesis 1: Convergent validity

Next, we examined the convergent validity of the SSI scale by examining the extent to which scores on the scale correlate with theoretically relevant variables. As in the previous studies, we predicted that the SSI would be correlated with political conservatism. The results supported this prediction (*ρ* = .40, *p* < .001).

We also predicted that men would score higher on the SSI scale than women. The results of an independent samples *t*-test confirmed this prediction. Consistent with the results from the previous studies, men endorsed the SSI significantly more strongly than women (see Table 8). Once again, the differences between men and women in both samples of Study 3 were between one-half and one point on the SSI scale, and the standard deviations were approximately one point. Therefore, it is important to note that the differences within men and within women were at least as large as the differences between men and women. Furthermore, the distributions of men’s and women’s scores overlapped quite a bit (see Fig 1).

#### Hypothesis 6: System justification

Finally, we used the SSI scale to test the novel prediction that participants who were exposed to system threat would endorse the separate spheres ideology more strongly than those who were not exposed to system threat. The results of an independent samples *t*-test confirmed this prediction (see Fig 2). In Study 3b, participants in the *system-threat* condition once again had significantly higher SSI scores (*M* = 3.28, *SD* = 1.04) than participants in the *no-threat* condition (*M* = 2.88, *SD* = 1.11, *t*(121) = 2.05, *p* < .05, *Cohen’s d* = .37). As in Study 3a, participants responded to system threat by strengthening their support for the idea that men and women should occupy separate spheres in society.

It should be noted that it is not yet clear whether system threat in Studies 3a and 3b caused SSI scores to rise, whether the no-threat stimuli caused SSI scores to fall, or whether a combination of these processes occurred. Evidence for the role of system threat in increasing SSI scores is that in both studies the mean SSI score for the system threat condition was equal to or higher than the highest mean score in all of the other samples (Study 2a). However, it is also possible that the no-threat stimuli caused SSI scores to fall; evidence for this interpretation is that in both studies, the mean score for the no-threat condition was lower than the lowest mean score in all of the other samples (Study 2c). Thus, it is likely that the threat stimuli increased SSI scores at the same time that the no-threat stimuli decreased SSI scores. Future research should examine this question using a third, neutral condition that neither threatens nor affirms the status quo. In any case, the results of Studies 3a and 3b provide robust evidence of motivated reasoning and support our contention that the SSI is a motivated, system-justifying ideology.

## General Discussion

We have presented the separate spheres ideology as a psychological construct characterized by individual differences and a motivated system-justifying function, operationalized the SSI with a new scale measure, and modeled the SSI as a predictor of important outcomes related to gender discrimination and inequality in society.

First, we developed the Separate Spheres Ideology scale, a reliable and valid measure of individuals’ support for the SSI. The SSI scale exhibited high reliability and consistent psychometric properties across all seven samples. This was no small feat, considering that the study included two student samples, three samples of diverse American adults, one sample of adults with jobs, and one sample of adults with supervisory responsibilities in their jobs. The reliability of the scale and the consistency of the results support the proposition of the Separate Spheres Model that endorsement of the SSI should be characterized as an individual difference.

Second, the SSI scale exhibited convergent validity and incremental predictive validity. SSI scores correlated in the predicted direction with a wide variety of contemporary, conceptually related measures. The SSI scale also predicted important attitudes and behaviors above and beyond the effects of existing measures in psychology. The SSI as a psychological construct and the SSI scale as a measure both seem to contribute to our understanding of gender inequality in ways not accomplished by current theoretical and measurement approaches to the study of gender attitudes.

Third, we provided support for the Separate Spheres Model contention that the SSI has important implications for gender inequality in society. We found that individuals’ endorsement of the SSI was related to stronger opposition to policies that would alleviate the caregiving pressures many women experience, allow men to derive more benefits from family life, and decrease economic disparities that disadvantage women (Hypothesis 2). Endorsement of the SSI was also related to supervisors’ discriminatory acts against employees with caregiving responsibilities (Hypothesis 5). These findings represent two types of gendered workplace outcomes that the Separate Spheres Model’s individual-difference approach is in a unique position to address, as the existing dominant approach to gendered workplace outcomes focuses on backlash toward individual targets’ gendered behavior. SSI scores also predicted gendered income distributions within families (Hypothesis 3), and they predicted individuals’ own reported participation in the work and domestic spheres in their everyday lives (Hypothesis 4). These are important real-world outcomes that go beyond simply predicting scale measures with other scale measures. These findings all demonstrate the substantive (and methodological) utility of the Separate Spheres Model.

Fourth, we provided experimental support for the novel hypothesis that the separate spheres ideology takes the functional form of a system-justifying ideology. System threat in two different forms caused participants to strengthen their support for the SSI. Expressing their separate spheres beliefs also seemed to serve a palliative function for participants, helping them to resolve their heightened need for system justification. The results of these studies suggest that the SSI is not simply a passive set of attitudes that individuals hold about proper gender roles, but is motivated and is actively used by participants to defend and justify the gendered status quo. Furthermore, these findings are particularly noteworthy, because the threats in question were not targeted to gender roles in any way. The threatening newspaper article in Study 3a discussed the social, economic, and political decline of the United States, and the threatening essay in Study 3b discussed the failure of the American Dream. Despite the fact that neither of these manipulations threatened traditional gender roles, participants responded to these threats by increasing their reliance on an ideology that justifies and promotes gendered segregation in society. This finding suggests that there are many opportunities in everyday environments to trigger system threat and increase individuals’ endorsement of the SSI.

Fifth, the results of these studies supporting the novel hypotheses of the Separate Spheres Model were characterized by ecological validity in terms of both the samples and the outcomes. Participants in five of the seven samples were adults who grapple with work-family decisions on a day-to-day basis. Study 2d in particular was comprised of individuals who have the power to make employment decisions about other workers, including approval for leave and other flexibility accommodations. Furthermore, the effects of the SSI were not limited to lab-based measures that may not generalize to real-world behaviors. Endorsement of the SSI predicted men’s and women’s gendered participation in the work and domestic spheres in their everyday lives, the reported income distribution within participants’ families, and supervisors’ self-reported discriminatory conduct in the workplace. These findings support the proposition of the Separate Spheres Model that the SSI has an important relationship to gendered inequality and suggest that this relationship is robust.

Finally, the results are consistent with previous research findings that have demonstrated that women often endorse traditional gender attitudes along with men. Particularly with forms of sexism that are nuanced and more positive in tone (such as benevolent sexism and the separate spheres ideology), women can and do espouse sexist beliefs nearly as often as men. Across the seven samples in this project, we found that while men exhibited slightly higher average scores than women, the distributions of men’s and women’s scores overlapped quite a bit. Perhaps this helps to explain the persistence of this belief system over time.

We have proposed that the separate spheres ideology regards the domestic and public spheres as equally valuable, even while de facto insisting that they remain segregated by gender. It is also the case, however, that the public sphere is more highly valued in financial terms in our society, with the majority of domestic work being unpaid. It is not yet clear whether individuals who endorse the SSI sincerely regard the two spheres as equally valuable to society or regard the public sphere as inherently more valuable but use separate-but-equal rhetoric as a way to uphold gendered institutions. Further research is needed to examine these possibilities.

Generally speaking, the results of these studies suggest that the Separate Spheres Model offers conceptual and methodological advantages to supplement existing approaches to the psychological study of gendered inequality. The prescriptive stereotyping and backlash approach has been extremely fruitful and provided much of the inspiration for this work. However, measuring gendered ideology as an individual difference and modeling it as a predictor of gendered outcomes allows psychologists to examine gendered outcomes in new ways and adds value to existing empirical approaches. The results of these studies demonstrate that individual and situational variation in endorsement of the SSI systematically predict outcomes that are consequential for gender inequality. This approach will allow social psychologists to investigate the antecedents and psychological processes underlying the separate spheres ideology and design new interventions to attenuate its harmful effects. These interventions may include strategies such as the de-biasing training used to weaken the effects of implicit associations (see, e.g., [58]), organizational factors to constrain how much SSI beliefs can manifest in workplace decision-making (see, e.g., [59]), message framing strategies that reduce system threat (see, e.g., [52]), or legal reforms designed to reduce discrimination [60–61].

Until now, social psychologists have not operationalized the SSI as an individual-differences measure. The potential uses of the SSI scale are numerous. For example, future research may examine the role of the SSI in workplaces, including the extent to which individual endorsement of the SSI interacts with structural aspects of the workplace and leads to greater discrimination and inequality within organizations. The Separate Spheres Model would predict that, under certain workplace conditions, supervisors’ endorsement of the SSI plays a role in flexibility stigma and family responsibilities discrimination. Researchers may also investigate endorsement of the SSI over the life course; early childhood exposure to gendered messages in the media and elsewhere may affect the development of this worldview. Researchers could examine the role of the SSI in political institutions; average support for the SSI in a district might decrease women’s emergence as political candidates. Finally, researchers may investigate more subtle priming effects on individuals’ endorsement of separate spheres, such as changes in SSI scores after exposure to sexist advertising. In sum, the Separate Spheres Model, which conceptualizes the SSI as an ideology characterized by individual differences and a system-justifying function, open doors for researchers looking to examine the psychological antecedents, processes, and consequences of endorsement of gendered spheres.

## Appendix A: The Separate Spheres Ideology Scale

1. Women can learn technical skills, but it doesn’t come as naturally as it does for most men.
2. If one person in a heterosexual marriage needs to quit working, it usually makes more sense for the husband to keep his job.
*3. Children with single parents can be just as well off as children with both a mom and a dad.
4. When it comes to voting for president, I’m more comfortable trusting a man to make tough political decisions than a woman.
*5. When a married couple divorces, judges shouldn’t assume that the mother is the more “natural” parent.
6. Most men naturally enjoy a tough and competitive career more than women do.
7. I would feel more comfortable if my auto mechanic was a man, rather than a woman.
*8. If we got rid of stereotyping and discrimination, differences between men and women would mostly disappear.
9. Women can learn how to be good leaders in the workplace, but it doesn’t come as naturally as it does for most men.
10. It’s natural for a woman to be fulfilled by taking care of her children, but most men feel better when they have a good career, too.
11. There are certain caregiving jobs, like nursing, that just naturally fit with women’s skills better than men’s skills.
12. Most kids are better off if their dad is the primary provider for the whole family.
*13. I would feel equally comfortable with a repair-man or a repair-woman to fix something in my house.
*14. It’s just as important to most women as it is to men to have a successful career.
15. When it comes to making tough business decisions, men tend to have special abilities that most women don’t have.

### Response scale

1. Strongly Disagree
2. Moderately Disagree
3. Slightly Disagree
4. Neither Agree nor Disagree
5. Slightly Agree
6. Moderately Agree
7. Strongly Agree

*Reverse-scored

## Supporting Information

S1 File Scree plots suggest a single dominant factor for SSI scale items in each sample (Studies 2 and 3). (PDF)

S2 File System Threat Manipulations (Study 3). (PDF)

## References

[1] Heilman M. Description and prescription: How gender stereotypes prevent women’s ascent up the organizational ladder. J Soc Issues. 2001;57: 657–674.

[2] Fuegen K, Biernat M, Haines E, Deaux K. Mothers and fathers in the workplace: How gender and parental status influence judgments of job-related competence. J Soc Issues. 2004;60: 737–754.

[3] Cuddy A, Fiske S, Glick P. When professionals become mothers, warmth doesn’t cut the ice. J Soc Issues. 2004;60: 701–718.

[4] Eagly A, Karau S. Role congruity theory of prejudice toward female leaders. Psychol Rev. 2002;109: 573–598. 12088246 10.1037/0033-295x.109.3.573

[5] Heilman M, Okimoto T. Motherhood: A potential source of bias in employment decisions. J Appl Psychol. 2008;93: 189–198. 10.1037/0021-9010.93.1.189 18211144

[6] Rudman L, Glick P. Prescriptive gender stereotypes and backlash toward agentic women. J Soc Issues. 2001;57: 743–762.

[7] Bowles H, Babcock L, Lai L. Social incentives for gender differences in the propensity to initiate negotiations: Sometimes it does hurt to ask. Organ Behav Hum Decis Process. 2007;103: 84–103.

[8] Heilman M, Wallen A, Fuchs D, Tamkins M. Penalties for success: Reactions to women who succeed at male gender-typed tasks. J Appl Psychol. 2004;89: 416–427. 15161402 10.1037/0021-9010.89.3.416

[9] Phelan J, Moss-Racusin C, Rudman L. Competent yet out in the cold: Shifting criteria for hiring reflect backlash toward agentic women. Psychol Women Q. 2008;32: 406–413.

[10] Williams J, Blair-Loy M, Berdahl J. Cultural schemas, social class, and the flexibility stigma. J Soc Issues. 2013;69: 209–234.

[11] Berdahl J, Moon S. Workplace mistreatment of middle class workers based on sex, parenthood, and caregiving. J Soc Issues. 2013;69: 341–366.

[12] Coltrane S, Miller E, DeHaan T, Stewart L. Fathers and flexibility stigma. J Soc Issues. 2013;69: 279–302.

[13] Rudman L, Mescher K. Penalizing men who request a family leave: Is flexibility stigma a femininity stigma? J Soc Issues. 2013;69: 322–340.

[14] Vandello J, Hettinger V, Bosson J, Siddiqi J. When equal isn’t really equal: The masculine dilemma of seeking work flexibility. J Soc Issues. 2013;69: 303–321.

[15] Coontz S. A strange stirring: The feminine mystique and American women at the dawn of the 1960s. New York: Basic Books, Inc; 2011.

[16] Hochschild A, Machung A. The Second Shift. New York: Viking Penguin; 1989.

[17] Greenstein T. Husbands’ participation in domestic labor: Interactive effects of wives’ and husbands’ gender ideologies. J Marriage Fam. 1996;58: 585–595.

[18] Kroska A. Conceptualizing and measuring gender ideology as an identity. Gend Soc. 2000;14: 368–394.

[19] Kroska A. Exploring the consequences of gender ideology-work discrepancies. Sex Roles. 2009;60: 313–338.

[20] Minnotte K, Minnotte M, Pederson D, Mannon S, Kiger G. His and her perspectives: Gender ideology, work-to-family conflict, and marital satisfaction. Sex Roles. 2010;63: 425–438.

[21] Brescoll V, LaFrance M. The correlates and consequences of newspaper reports of research on sex differences. Psychol Sci. 2004;15: 515–520. 15270995 10.1111/j.0956-7976.2004.00712.x

[22] Martin C, Parker S. Folk theories about sex and race differences. Pers Soc Psychol Bull. 1995;21: 45–57.

[23] Prentice D, Miller D. Essentializing differences between men and women. Psychol Sci. 2006;17: 129–135. 16466420 10.1111/j.1467-9280.2006.01675.x

[24] Eagly A. Sex differences in social behavior: A social-role interpretation. Hillsdale: Lawrence Erlbaum Associates; 1987.

[25] Gerson K. The unfinished revolution: How a new generation is reshaping family, work, and gender, in America. New York, NY: Oxford University Press, Inc; 2010.

[26] Pedulla D, Thébaud S. Can we finish the revolution? Gender, work-family ideals, and institutional constraint. Am Sociol Rev. 2015;80: 116–139. 26365994 10.1177/0003122414564008 PMC4564125

[27] Kuperberg A, Stone P. The media depiction of women who opt out. Gend Soc. 2008;22: 497–517.

[28] Williams J, Manvell J, Bornstein S. “Opt out” or pushed out?: How the press covers work-family conflict. 2006. In: The Center for WorkLife Law. Available: http://www.worklifelaw.org/pubs/OptOutPushedOut.pdf.

[29] North A. Why the opt-out story won’t die. Buzzfeed. 18 Mar 2013. Available: http://www.buzzfeed.com/annanorth/why-the-opt-out-story-wont-die. Accessed 28 Apr 2015.

[30] Saad L. Stay-at-home moms in U.S. lean independent, lower-income. 2012 Apr 19. In: Gallup. Available: http://www.gallup.com/poll/153995/Stay-Home-Moms-Lean-Independent-Lower-Income.aspx?utm_source=alert&utm_medium=email&utm_campaign=syndication&utm_content=morelink&utm_term=All%20Gallup%20Headlines.

[31] Stone P, Hernandez L. The all-or-nothing workplace: Flexibility stigma and “opting out” among professional-managerial women. J Soc Issues. 2013;69: 235–256.

[32] Swim J, Aikin K, Hall W, Hunter B. Sexism and racism: Old-fashioned and modern prejudices. J Pers Soc Psychol. 1995;68: 199–214.

[33] Glick P, Fiske S. The Ambivalent Sexism Inventory: Differentiating hostile and benevolent sexism. J Pers Soc Psychol. 1996;70: 491–512.

[34] Glick P, Fiske S. An ambivalent alliance: Hostile and benevolent sexism as complementary justifications for gender inequality. Am Psychol. 2001;56: 109–118. 11279804

[35] Jost J, Kay A. Exposure to benevolent sexism and complementary gender stereotypes: Consequences for specific and diffuse forms of system justification. J Pers Soc Psychol. 2005;88: 498–509. 15740442 10.1037/0022-3514.88.3.498

[36] Spence JT, Helmreich R, Stapp J. A short version of the Attitudes toward Women Scale (AWS). Bull Psychon Soc. 1973;2: 219–220.

[37] Twenge J. Attitudes toward women, 1970–1995. Psychol Women Q. 1997;21: 35–51.

[38] Davis S, Greenstein T. Gender ideology: Components, predictors, and consequences. Annu Rev Sociol. 2009;35: 87–105.

[39] Epstein S. The stability of behavior: I. On predicting most of the people much of the time. J Pers Soc Psychol. 1979;37: 1097–1126.

[40] Furr M. Scale construction and psychometrics for social and personality psychology. London: SAGE Publications Ltd; 2011.

[41] Jost J, Glaser J, Kruglanski A, Sulloway F. Political conservatism as motivated social cognition. Psychol Bull. 2003;129: 339–375. 12784934 10.1037/0033-2909.129.3.339

[42] Crosby F. Juggling: The unexpected advantages of balancing career and home for women and their families. New York: The Free Press; 1991.

[43] Williams J, Bornstein S. The evolution of “FReD”: Family responsibilities discrimination and developments in the law of stereotyping and implicit bias. Hastings Law J. 2008;59: 1311–1358.

[44] Jost J, Liviatan I, van der Toorn J, Ledgerwood A, Mandisodza A, Nosek B. System justification: How do we know it’s motivated? In: Bobocel D, Kay A, Zanna M, Olson J, editors. The psychology of justice and legitimacy: The Ontario symposium. Vol. 11 Hillsdale: Erlbaum; 2011 pp. 173–203.

[45] Kay A, Jost J, Young S. Victim derogation and victim enhancement as alternate routes to system justification. Psychol Sci. 2005;16: 240–246. 15733206 10.1111/j.0956-7976.2005.00810.x

[46] Wakslak C, Jost J, Bauer P. Spreading rationalization: Increased support for large-scale and small-scale social systems following system threat. Soc Cogn. 2011;29: 288–302.

[47] Brescoll V, Uhlmann E, Newman G. The effects of system-justifying motives on endorsement of essentialist explanations for gender differences. J Pers Soc Psychol. 2013;105: 891–908. 10.1037/a0034701 24295379

[48] Moss-Racusin C, Phelan J, Rudman L. When men break the gender rules: Status incongruity and backlash against modest men. Psychol Men Masc. 2010;11: 140–151.

[49] Rudman L, Moss-Racusin C, Phelan L, Nauts S. Status incongruity and backlash effects: Defending the gender hierarchy motivates prejudice against female leaders. J Exp Soc Psychol. 2012;48: 165–179.

[50] Jost J, Chaikalis-Petritsis V, Abrams D, Sidanius J, van der Toorn J, Bratt C. Why men (and women) do and don’t rebel: Effects of system justification on willingness to protest. Pers Soc Psychol Bull. 2012;38: 197–208. 10.1177/0146167211422544 21911420

[51] Wakslak C, Jost J, Tyler T, Chen E. Moral outrage mediates the dampening effect of system justification on support for redistributive social policies. Psychol Sci. 2007;18: 267–274. 17444925 10.1111/j.1467-9280.2007.01887.x

[52] Feygina I, Jost J, Goldsmith R. System justification, the denial of global warming, and the possibility of “system-sanctioned change”. Pers Soc Psychol Bull. 2010;36: 326–338. 10.1177/0146167209351435 20008965

[53] MacKinnon D, Krull J, Lockwood C. Equivalence of the mediation, confounding, and suppression effect. Prev Sci. 2000;1: 173–181. 11523746 10.1023/a:1026595011371 PMC2819361

[54] Paulhus D, Robins R, Trzesniwski K, Tracy J. Two replicable suppressor effects in personality research. Multivariate Behav Res. 2004;39: 301–326. 10.1207/s15327906mbr3902_7 26804578

[55] Shrout P, Bolger N. Mediation in experimental and non-experimental studies: New procedures and recommendations. Psychol Bull. 2002;7: 422–445. 12530702

[56] Berinsky A, Huber G, Lenz G. Evaluating online labor markets for experimental research: Amazon.com’s Mechanical Turk. Political Analysis. 2012;20: 351–368.

[57] Buhrmester M, Kwang T, Gosling S. Amazon.com’s Mechanical Turk: A new source of inexpensive, yet high-quality, data? Perspect Psychol Sci. 2011;6: 3–5. 10.1177/1745691610393980 26162106

[58] Correll J, Park B, Judd C, Wittenbrink B, Sadler M, Keesee T. Across the thin blue line: Police officers and racial bias in the decision to shoot. J Pers Soc Psychol. 2007;92: 1006–1023. 17547485 10.1037/0022-3514.92.6.1006

[59] Biernat M, Fuegen K, Kobrynowicz D. Shifting standards and the inference of incompetence: Effects of formal and informal evaluation tools. Pers Soc Psychol Bull. 2010;36: 855–868. 10.1177/0146167210369483 20495094

[60] Miller A. The separate spheres ideology: An improved empirical and litigation approach to family responsibilities discrimination. Minn Law Rev. 2014;99: 343–379.

[61] Miller A. The use (and misuse) of the same actor inference in family responsibilities discrimination litigation: Lessons from social psychology on flexibility stigma. William Mitchell Law Rev. 2015;41: 1032–1089.

