# Supplementary material for: The Separate Spheres Model of Gendered Inequality
Source: PLoS One. 2016 Jan 22;11(1):e0147315. doi: 10.1371/journal.pone.0147315 (PMC4723260; doi:10.1371/journal.pone.0147315)

S1 File. Scree plots suggest a single dominant factor for SSI scale items in each sample (Studies 2 and 3).

Study 2a

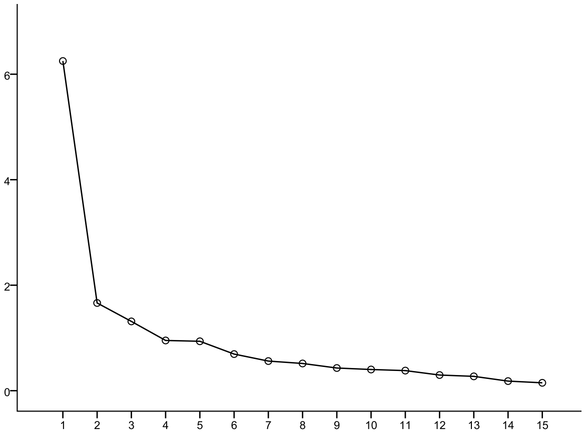

Study 2b

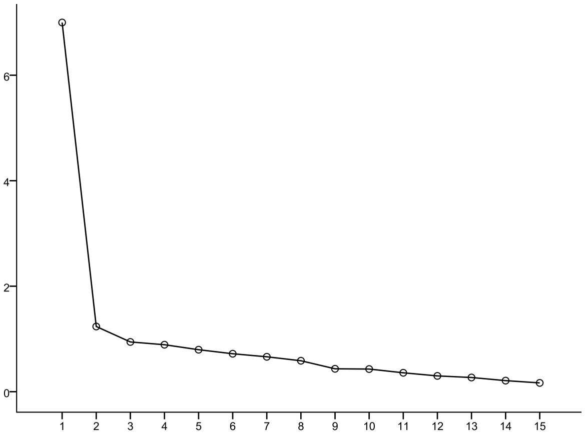

Study 2c

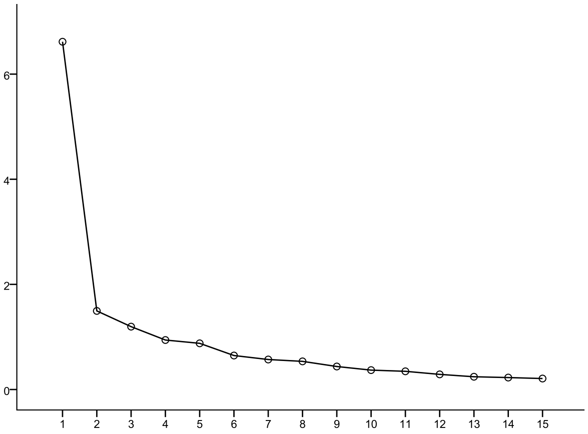

Study 2d

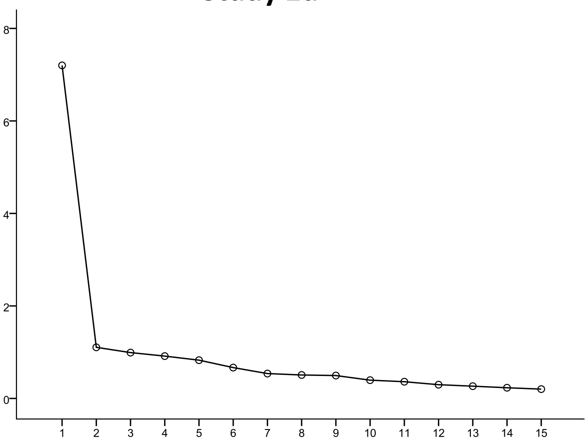

Study 3a

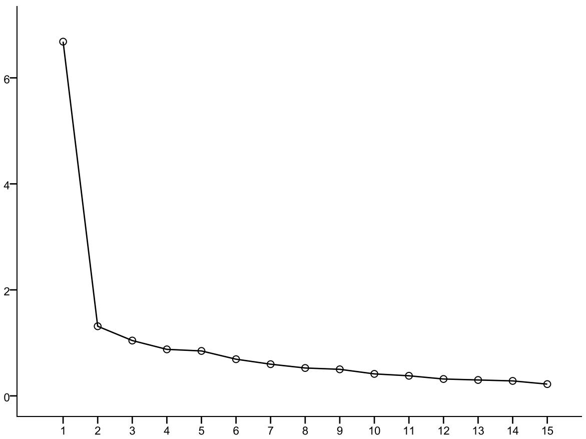

Study 3b

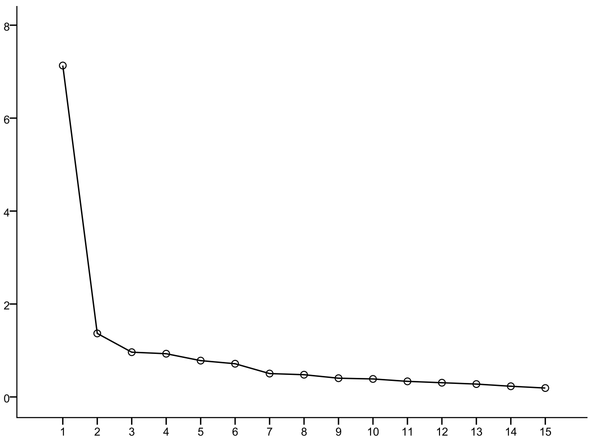

Supplement: S1 File — (PDF) [file pone.0147315.s001.pdf]
