# Supplementary material for: The Separate Spheres Model of Gendered Inequality
Source: PLoS One. 2016 Jan 22;11(1):e0147315. doi: 10.1371/journal.pone.0147315 (PMC4723260; doi:10.1371/journal.pone.0147315)
Supplement: S2 File — (PDF) [file pone.0147315.s002.pdf]

S2 File: System Threat Manipulations (Study 3)

The following stimuli were used to manipulate system threat in Study 3. Differences between the conditions in each Study are indicated with italics.

Study 3a (stimuli taken from Kay, Jost, & Young, 2005):

System-threat condition:

These days, many people in the United States feel *disappointed with the nation's condition*. Many citizens feel that the country *has reached a low point* in terms of social, economic, and political factors. It seems that many countries in the world *are enjoying better* social, economic, and political conditions *than* the U.S. *More and more* Americans express a willingness to leave the United States and emigrate to other nations.

No-threat condition:

These days, *despite the difficulties the nation is facing*, many people in the United States feel *safer and more secure relative to the past*. Many citizens feel that the country *is relatively stable* in terms of social, economic, and political factors. It seems that *compared with* many countries in the world *the* social, economic, and political conditions *in* the U.S. *are relatively good*. *Very few* Americans express a willingness to leave the United States and emigrate to other nations.

Study 3b:

System-threat condition:

I read an article recently that said a large percentage of low-income students in the United States *aren't* able to go to college. Since the *recession hit*, more and more low-income students *can't*

afford to go to college, *even if* their grades are good enough to be accepted. Sometimes I *worry* that I'm going to be one of those students. When I look at my life, it feels like the American Dream they teach you about in school *doesn't* really exist. I am the only person I know at my school who has to work long hours to support their family, and I know that this experience has shaped the person I am now. I've been working in some way or another to help my family for as long as I can remember. When I was 11, my parents worked opposite shifts, so they were barely around to see each other or me and my younger brother. I had to watch my brother every day after school while my friends got to go outside and play. When I was 16, I got my first paid job, and I've done my best to contribute as much money as I can to the family. My parents each work full time trying to make ends meet, but we still seem to always need the extra bit of money that my job brings in at the end of the month. I had to give up many of the small things in life that my friends take for granted, like music lessons or name-brand shoes. It's *hard* knowing that *no matter how many hours* my parents and I *work*, we will *never* be able to afford a college education for me *by ourselves*.

<u>No-threat condition:</u>

I read an article recently that said a large percentage of low-income students in the United States *are* able to go to college. Since the *economic recovery began*, more and more low-income students *can* afford to go to college *as long as* their grades are good enough to be accepted. Sometimes I *feel really grateful* that I'm going to be one of those students. When I look at my life, it feels like the American Dream they teach you about in school really *does* exist. I am the only person I know at my school who has to work long hours to support their family, and I know that this experience has shaped the person I am now. I've been working in some way or another

to help my family for as long as I can remember. When I was 11, my parents worked opposite shifts, so they were barely around to see each other or me and my younger brother. I had to watch my brother every day after school while my friends got to go outside and play. When I was 16, I got my first paid job, and I've done my best to contribute as much money as I can to the family. My parents each work full time trying to make ends meet, but we still seem to always need the extra bit of money that my job brings in at the end of the month. I had to give up many of the small things in life that my friends take for granted, like music lessons or name-brand shoes. It's *rewarding* knowing that *because* my parents and I *worked so hard*, we will be able to afford a college education for me.
